# Phenotypic and genetic characterization of sixteen grain and dual-type industrial hemp varieties (*Cannabis sativa* L.) for agronomic and yield component traits

**DOI:** 10.3389/fpls.2025.1632346

**Published:** 2025-10-20

**Authors:** Kusum Raj Tamang, Thomas P. Mawhinney, Christian B. Carson, Joshua Yeboah Asiamah, Sakina Haruna Mahdi, Emily Reed, Swastika Sharma, Prabesh Koirala, Jaimin Patel, Clement Akotsen Mensah, Babu Valliyodan

**Affiliations:** ^1^ Department of Agriculture and Environmental Sciences, Lincoln University of Missouri, Jefferson City, MO, United States; ^2^ Department of Biochemistry, University of Missouri, Columbia, MO, United States; ^3^ IR-4 Project Headquarters, North Carolina State University, Raleigh, NC, United States; ^4^ Alabama Cooperative Extension System, Alabama A&M and Auburn Universities, Normal, AL, United States

**Keywords:** industrial hemp, grain hemp, dual-type hemp, grain yield, flowering trait, seed shattering resistance, seed composition

## Abstract

Industrial hemp (*Cannabis sativa* L.) is a multipurpose crop primarily grown for fiber, grain, and cannabinoids. Due to its high-quality protein and oil composition, industrial hemp grain is increasingly an important crop for a nutritional source. Although the global demand for hemp grain is increasing, research exploring the genetic and climatic effects on agronomic and seed composition traits is limited. Furthermore, there has been very little research conducted on seed development and shattering. Therefore, to study this biological phenomenon and for optimal hemp grain production in Missouri, suitable cultivars for this production region were compared to identify the best-suited ones. Key physiological and seed compositional traits were studied. We found significant variation in plant height, diameter, biomass, grain yield, crude protein, and crude fat among the evaluated varieties. The dual type variety, Futura 83, showed superior performance and yield and has many suitable traits for the Missouri production region, with the grain yield ranging from 2434–2793 kg/ha. In addition, we studied the expression of two candidate genes associated with seed shattering resistance and flowering. Both genes were expressed differentially among various hemp tissues. The expression of the *GmPdh1* homolog gene was higher in mature seeds, and the *GmDt1* homolog was higher in flower tissues, suggesting their potential role in seed dispersal and flowering. However, further research is required for functional validation and increasing the crop yield and seed composition.

## 1 Introduction

Industrial hemp (*Cannabis sativa* L.) is a versatile crop grown for fiber, seeds, and flowers (Deguchi et al., 2020; Vasantha Rupasinghe et al., 2020; Muedi et al., 2024). Legally, they are defined as the cannabis plant that has less than 0.3 percent Δ-9 tetrahydrocannabinol (THC) (Agriculture Improvement Act, 2018). Use of this plant is significant across multiple sectors, including agriculture, manufacturing, healthcare, and biofuel (Gülizar et al., 2025).

Further, they are categorized based on their primary use (Deguchi et al., 2020; Vasantha Rupasinghe et al., 2020). The four main types are fiber-type, grain-type, dual-type, and cannabinoid-type, which are characterized by their distinct plant phenotypes and uses. Fiber-type hemp is primarily grown for its fibers (Islam and Hasan, 2025). Similarly, grain-type hemp is cultivated for its grain, dual-type hemp, which is used for both grain and fiber production, while cannabinoid hemp is grown for its cannabinoid and other secondary metabolites (ElSohly et al., 2017; Das et al., 2020; Muedi et al., 2024).

Industrial hemp has nine primary growth phases: seed germination and emergence, leaf growth, lateral shoot formation, stem elongation, first flowering, full flowering, fruit development, fruit ripening, and senescence (Mishchenko et al., 2017; Petit et al., 2020a; Rahemi et al., 2021).

Grain yield is the key trait for grain and dual-type hemp varieties. The seeds are a nutrient-packed source of excellent protein that ranges from 24.20 to 30%. The protein content in hemp is higher than that of chickpea and on par with that of soybeans, which are commonly used plant-based proteins (Liu et al., 2023). In addition to proteins, they are a good source of saturated and polyunsaturated fatty acids. Likewise, they are also widely used in the cosmetic industry (Callaway, 2004; Cattaneo et al., 2021).

Although some industrial hemp varieties are monoecious, most of the hemp varieties are dioecious, having both separate male and female flowers (Hall et al., 2012; Petit et al., 2020a; Toth et al., 2020; Cattaneo et al., 2021). Flowering is a complex trait in hemp that is influenced by numerous genes and their interactions (Ma, 2005; Liu et al., 2010; Petit et al., 2020a). The candidate genes that sense and transmit light signals in the flowering pathway include *cry1* (cryptochrome), *phyA* and *phyE* (phytochromes), spa1 (a regulator of *phyA*), *uvr8* (a UV-B receptor), and *xap5* (a circadian regulator of light responses). Similarly, several transcription factors such *as bed, bZIP, constans, floricaula, leafy, flowering locus D, flowering locus T, MADS-box genes, squamosa*, and *vrn1* are also related to flowering pathways (Petit et al., 2020). The circadian clock gene *CsPRR37* in auto-flowering cannabis. Likewise, CsFT1 (LOC115700781), an ortholog of the Arabidopsis *FLOWERING LOCUS T* gene, was identified as a candidate gene controlling photoperiod-insensitive flowering in *Cannabis sativa* (Dowling et al., 2024). Similarly, circadian clock gene *CsPRR37* is also related to flowering time in *Cannabis sativa* particularly as its role in repressing the FT gene (Leckie et al., 2024). In addition to these genes, one of the essential flowering genes is *Centroradialis-like protein (CENLP)* that causes shoot growth and flowering (Amaya et al., 1999). The *Cannabis sativa CEN-like protein 2* (LOC115720824) is homologous to *Glycine max Terminal Flower 1b* (*GmTFL1 b).* The function of *GmTFL1 b* varies in different plant species. The function of the *TFL1 gene* is the suppression of flowering by repressing the role of florigen FT, which is responsible for the transition to the reproductive phase from the vegetative phase (Wickland and Hanzawa, 2015). In soybeans, the gene *GmTFL1 b* is a key regulator of stem termination, influencing whether the plant exhibits a determinate or indeterminate growth habit (Quach and Nguyen, 2015; Mohamedikbal et al, 2024). Additionally, knockout mutants *tfl1c/tfl1d* flowered earlier than the wild-type plants (Wang and Li, 2008). Similarly, in Arabidopsis, the *TFL1* gene is the homolog of the *GmTFL1b* gene. In the *tfl1 mutant*, genes promoting floral identity are expressed prematurely, leading to early flowering and showing conversion of the shoot apical meristem into a terminal flower (Hanano and Goto, 2011). While several photoperiodism and flowering genes have been previously characterized in hemp, such as *CsFT1*, *CsPRR37*, and *Autoflower1*, our study focused on *CsCENLP* to explore an additional gene. Investigating this gene allows us to complement existing knowledge and uncover potentially new genes that regulate flowering time. Furthermore, qPCR enables us to directly assess whether its expression patterns correlate with flowering phenotypes.

While seed shattering is an essential trait for natural seed dispersion, it is one of the major issues in industrial hemp, where it reduces the total grain yield by 40-80% (Rahemi et al., 2021). A complex network of interacting genes governs seed dispersal. Generally, this phenomenon is a dominant trait in plants such as rice, wheat, cowpea, and turnip rape. In sorghum, the key shattering gene encodes a YABBY transcription factor. Its homologous genes in rice cause seed shattering by forming an abscission layer between the pedicel and spikelet (monocots) (Maity et al., 2021). In soybeans, seed shattering is closely linked to the dehiscence zone (DZ) characteristics between cells in the seed pod valve. Seed shattering is primarily driven by the loss of adhesion between cells in DZ. This loss of adhesion is influenced by two key enzymes: polygalacturonase (PG) and cellulase (CE). PG breaks down the galacturonan chains of pectin, while CE targets cytoskeletal elements in the cell wall, facilitating the breakdown of the middle lamella (Marsh et al., 2023). *GmPdh1* is one of the key genes responsible for seed shattering because the pod abscission layer of homozygous loss-of-function *pdh1* mutants remains intact longer, significantly reducing seed shattering (Funatsuki et al., 2014). We selected *GmPdh1* because of its well-characterized, tissue-specific role in regulating lignin deposition and its direct effect on pod shattering in soybean. Given the similarity of the shattering process in hemp, we hypothesize that *Pdh1* may play a comparable role in seed shattering.

The global industrial hemp market value is anticipated to increase and is expected to achieve a market valuation of $36 billion by 2026 (Dhoubhadel, 2021). Although grain and dual-type hemp have tremendous potential across multiple sectors, one of the major bottlenecks is the lack of reliable varieties for regional cultivation. Furthermore, most hemp varieties are imported from Europe, Canada, and China. And the lack of regional testing of the U.S.-based varieties presents another major challenge since there is climatic dissimilarity between these agroclimatic regions. Thus, there is a dire need for thorough varietal screening for selection (Wimalasiri et al., 2021). Hence, our primary goal is to identify suitable hemp varieties for Missouri and the Midwest production region.

## 2 Materials and methods

We compared the morphological traits of grain-type and dual-purpose hemp varieties, using a fiber-type variety as a positive control, which has been named the check variety in the results section. The check fiber variety was based on the previous studies conducted in the other part of the United States and the own Field Trial at Lincoln University of Missouri (Cornell Hemp Report, 2020). Since this is an early-stage experiment for the Missouri production region, we used two replications in this initial study and continued the research over a period of three years to assess consistency and performance.

### 2.1 Plant materials

We selected nine varieties in 2022 based on their suitability in other parts of the United States and their global performance (Rahemi et al., 2021; Cornell Hemp report, 2020; Ferfuia et al., 2021). The varieties selected can be found in **Table 1**. In 2023, a total of 16 varieties were screened, and the details of origin and use can be found in **Table 2**. Likewise, their flowering and maturation times can be found in **Table 3**. Likewise, for the planting season 2024, 10 varieties were selected for the varietal screening. The selection of the varieties was based on the previous year’s field performance and the availability of seeds for the planting season. Similarly, naturally growing (wild-type) American ferals were collected from different parts of Missouri. Their seeds were sorted and grown in the field. Among these, LHV-17, one of the best-adapted wild types, was selected for comparative studies. LHV-17 was then compared with domesticated varieties.

**Table 1:** Three-year comparisons of key morphological traits in industrial hemp varieties.

Variety	Plant standing (1-5)	Plant standing (1-5)	Plant standing (1-5)	Plant height (cm)	Plant height (cm)	Plant height (cm)	Stem diameter (mm)	Stem diameter (mm)	Stem diameter (mm)
	2022	2023	2024	2022	2023	2024	2022	2023	2024
Altair	2.25^cd^	3.75^bcde^	NA	115.13^de^	91.44^cdef^	NA	6.21^cd^	6.22^bc^	NA
Bialobrzeskie	3.5^abcd^	4.00^abcde^	4.00^ab^	109.42^de^	118.49^bcdef^	173.48^bcd^	5.43^cd^	5.51^c^	6.14^cd^
Carmaecta	NA	4.25^abcd^	4.50^a^	NA	172.72^ab^	286.26^a^	NA	7.90^bc^	9.54^bcd^
Felina	4.75^ab^	2.25^f^	NA	129.74^cde^	72.50^ef^	NA	5.49^cd^	4.58^c^	NA
Ferimon	4.5^abc^	2.25^f^	NA	123.82^cde^	77.47^ef^	NA	6.88^cd^	5.14^c^	NA
Fibror 79	3.25^abcd^	3.5^cde^	2.50^a^	168.47^cd^	152.4^bc^	175.26^bc^	6.381^cd^	6.40^bc^	8.99^bcd^
Futura 83	4.25^abc^	4.75^ab^	4.75^a^	172.72^cd^	161.29^ab^	221.23^ab^	8.56^bcd^	8.15^bc^	9.09^bcd^
Gravity	3.00^abcd^	4.5^abc^	NA	153.23^cde^	161.92^ab^	NA	11.44^abcd^	7.13^bc^	NA
Henola	2.50^bcd^	4.25^abcd^	3.75^ab^	67.31^e^	87.63^def^	136.85^c^	4.71^d^	4.41^c^	6.81^cd^
BVL3	5.00^a^	4.25^abcd^	4.00^ab^	213.76^bc^	164.69^ab^	200.66^bc^	9.70^abcd^	10.17^b^	10.66^bc^
Jinma	5.00^a^	5.00^a^	5.00^a^	298.01^ab^	224.53^a^	277.62^a^	16.51^ab^	16.91^a^	17.63^a^
BVL1	3.50^abcd^	3.00^ef^	3.75^ab^	147.32^cde^	89.90^cdef^	180.34^bc^	9.11^abcd^	5.33^c^	5.17^d^
BVL2	4.00^abcd^	3.25^defg^	NA	123.59^cde^	82.55^def^	NA	5.95^cd^	6.31^bc^	NA
Orion	4.00^abcd^	3.75^bcde^	NA	134.62^cde^	145.41^bcd^	NA	5.34^cd^	6.55^bc^	NA
Santhica	4.00^abcd^	3.25^def^	NA	137.99^cde^	133.35^bcde^	NA	7.64^bcd^	5.84^c^	NA
Uso 31	3.5^abcd^	2.25^f^	NA	111.76^de^	72.39^ef^	NA	4.70^d^	4.53^c^	NA
Vega	2.5^bcd^	2.25^f^	3.75^ab^	87.63^e^	68.16^f^	142.36^c^	4.32^d^	5.66^c^	7.34^cd^
F value	6.43	26.88	5.75	22.71	17.08	15.21	6.92	16.34	17.7380
P value	<0.001	<0.001	<0.01	<0.001	<0.001	<0.001	<0.001	<0.001	<0.001
Significance	***	***	**	***	***	***	***	***	***

ns, non-significant.

*Significant at p ≤ 0.05.

**Significant at p ≤ 0.01.

***Significant at p ≤ 0.001.

Values followed by different lowercase letters within a row are significantly different at P< 0.05 (Tukey’s HSD test). “NA” means those varieties were not planted in that year. Jinma, a fiber variety, was used as a check variety for comparison.

**Table 2:** Name of the variety along with its origin and use.

S.N	Variety	Origin	Use
1	Altair	Canada	Grain
2	Bialobrzeskie	Poland	Dual
8	BVL1	United States	Grain
9	BVL2	United States	Grain
16	BVL3	United States	Dual
12	Carmenecta	Italy	Dual
3	Felina	France	Dual
4	Ferimon	France	Dual
13	Fibror 79	France	Dual
14	Futura 83	France	Dual
15	Gravity	United States	Dual
11	Henola	Poland	Dual
10	Jinma	China	Fiber
5	Orion	France	Grain
17	Santhica	France	Dual
6	Uso 31	Ukraine	Grain
7	Vega	Canada	Grain

Jinma, a fiber variety, was used as a check variety for the comparison.

**Table 3:** Comparison of flowering and maturity time among industrial hemp varieties.

Variety	Days to male plant flowering	Days to female/(Monoecious) plant flowering	Days to maturity	Flowering type	Flowering status
Altair	NA	40	60	100% monoecious	Early flowering
Bialobrzeskie	NA	40	70	100% monoecious	Early flowering
BVL1	50	60-65	120	50% female flowers	Mid flowering
BVL2	50	60-65	115	50% female flowers	Mid flowering
BVL3	50	65-70	115	51.5% female flowers	Mid flowering
Carmenecta	50	65-70	120	72.5% Female flower	Mid flowering
Felina	NA	40-45	70	100% monoecious	Early flowering
Ferimon	NA	40-45	70	100% monoecious	Early flowering
Fibror 79	NA	45-50	75	100% monoecious	Early-Mid flowering
Futura 83	Na	65-70	120	100% monoecious	Mid flowering
Gravity	55	60-65	115	70.5% female flowers	Mid flowering
Henola	NA	40-45	75	100% monoecious	Early flowering
Jinma	90	105	140	80% Female flower	Late flowering
Orion	NA	40-45	75	100% monoecious	Early flowering
Santhica	NA	60-65	110	100% monoecious	Mid flowering
Uso 31	NA	40	55	100% monoecious	Early flowering
Vega	NA	40	60	100% monoecious	Early flowering

Jinma, a fiber variety, was used as a check for comparison.

### 2.2 Land preparation and planting

The field experiments were conducted at the George Washington Carver Farm, Lincoln University, Jefferson City, Missouri, in 2022, 2023, and 2024. The site is located at an elevation of 213 meters above sea level. The experiment was conducted using a Randomized Complete Block Design (RCBD) experimental layout in two replications. Because this was an early-stage, multi-year study to screen hemp varieties, using two replications was considered sufficient (Kempton and Gleeson, 1997). Planting was carried out using a Great Plains 1006 NT No-Till Compact Drill, with a plot size of 185.89 m² and row spacing set at 19.15 cm. Fertilizer was applied at a ratio of 75:20:30 nitrogen: phosphorus: potassium (N: P: K). Seeds were planted at a depth of 0.63 cm.

### 2.3 Field data collection

Weekly field data were collected for each season as indicated in **Table 4**.

**Table 4:** Summary of the methods for phenotypic data collection.

S. N	Traits studied	Unit	Methodology	Study significance
1	Germination percentage	%	It was calculated in Greenhouse conditions in a seed germination tray. Germination percentage (%) = $\frac{Plant\;germinated\;}{Seed\;sown}$ *100%	It helps to assess seed viability under a controlled environment.
2	Emergence percentage	%	It was calculated from a square meter plot in the field condition. Emergence (%) = $\frac{Plant\;emerged\;}{Seed\;sown}$ *100%	It helps to study the viability of the seed in the field conditions.
3	Plant height	cm	Weekly plant height was recorded from the soil surface to the apical tip using a measuring ruler.	It is an indicator of vegetative growth and plant vigor.
4	Plant Population	plants/m^2^	Plant population is the number of plants in a square meter at the maturation stage, counted using a square meter quadrant in each plot.	It is used in biomass prediction and modeling for yield-related traits.
5	Plant standing	Visual estimate (0–5 scale)	It is a visual score to assess the vigor of the plant and was noted based on the visual scoring on a scale of 0 to 5. 0: There was no plant establishment in the field 1-2: The plants had poor vigor with significant stunting and chlorosis 3: The plants had moderate growth with mild signs of stress 4: The plants had healthy growth, minor variation in height, and mild signs of stress 5: The plants had vigorous growth and uniform height and displayed resistance to common biotic pressures such as weeds, pests, and diseases	It helps to assess the vigor of the plant.
6	Flowering	Days	Male and female flowering times were noted when the flowers were seen. Early, intermediate, and late flowering was categorized on the basis of the time it takes to flower. Days to flower =Date of flowering- Date of sowing	Flowering is associated with yield.
7	Days to maturation	Days	It was measured based on the days the seeds reach a 20-30% moisture content from the germination stage. The seed moisture was calculated using the Mettler Toledo HC103 Halogen Moisture Analyzer.	It is important for harvest planning.
8	Female flower percentage	%	One hundred plants were randomly counted, and the number of female plants in those 100 plants were noted. Female flower percentage = $\frac{Number\;of\;female\;plants\;}{Total\;number\;of\;counted\;plants}$ *100%	It is important for yield and breeding applications.
9	Stomatal conductance (SC)	mol m^-^² s^-^¹	Stomatal conductance was measured from the young leaves using the LI 600 user manual (LICOR, Nebraska). Three plants were randomly selected from each replicate during the maturation stage before flowering. The linear regression analysis was performed to examine the relationship between stomatal conductance (gs) and independent variables such as vapor pressure deficit and transpiration rate using the lm model in R Studio.	SC is one of the significant determinants of photosynthesis.
10	Weed Infestation	Visual estimate (0–5 scale)	It was noted based on visual scoring on a scale of 0 to 5, 0: No weed infestation in the plot 1-2: Mild infestation, the plants outperformed the weeds 3-4: Moderate to noticeable infestation, the plants struggled to compete against the weeds 5: Severe infestation with clear signs of plant stress and decline Weed infestation percentage of plot = $\frac{Rating\;of\;the\;plot\;\left(0-5\right)\;}{5}$ *100%	It is an indicator of field competitiveness and management needs.
11	Seed shattering	Visual estimate (0–5 scale)	It was noted based on visual scoring on a scale of 0 to 5, 0 – No visible seed shattering. 0-1 – Very low shattering. 2-3 – Moderate shattering. Noticeable seed loss after gentle shaking. 3-4 – High shattering. Significant seed loss even in gentle touch. 4-5 – Extreme shattering. Nearly all seeds (>90%) are lost before or at harvest; very few remain on the plant.	
11	Grain yield	kg/hectare	Hemp grains were hand-harvested from a square meter plot in each plot and converted into kg/ha. The seed was dried in the shade and threshed using the Almaco bean thresher.	It is an important economic trait.
12	Diameter	mm	Diameter was recorded before harvesting using a vernier caliper	It is used for biomass prediction/Modelling.
13	Whole plant biomass	g	Before harvesting, five plants were randomly uprooted from each plot to a depth of 25–30 cm. The root debris was removed, the fresh weight of each plant was recorded, and the bags were labeled. Subsequently, these plant samples were kept in a drying room at 170°F for one week, after which their dry weights were recorded. Whole plant biomass = Root biomass + Stem biomass+ Flower biomass+ Leaves biomass	This trait is used for carbon modeling and biomass allocation studies.
14	Above-ground and below-ground biomass	g	Three representative plants were excavated to a depth of 25–30 cm. The above-ground and below-ground plant parts were separated by cutting the plants approximately 3–4 cm above the root. The fresh weights of the above-ground and below-ground plant parts were measured sequentially, followed by the determination of their respective dry weights, as mentioned in point 13. Above biomass =Whole plant biomass- Root biomass Below-ground = biomass Root biomass	This trait is important for carbon modeling and biomass allocation studies.

The table entails the nature of the data, unit, detailed methodology, and significance.

### 2.4 Seed compositional analysis

#### 2.4.1 Proximate analysis

A total of 25 g of seeds were harvested from each plot, with three technical replicates per treatment. Seeds were cleaned to remove debris and sent to the University of Missouri Agricultural Experiment Station Chemical Laboratories (ESCL) for proximate composition analysis. Crude protein was determined using AOAC Method 984.13 (A–D, 2006), crude fat using AOAC Method 920.39 (A), crude fiber using AOAC Method 978.10 (2006), and ash content using AOAC Method 942.05.

#### 2.4.2 Fractionation and quantification of seed storage proteins

200 mg of seeds were ground using liquid nitrogen in a pre-chilled mortar and pestle. Total and soluble proteins were extracted following the Osborne fractionation method (Osborne, 1924), separating proteins into water-soluble (albumins), salt-soluble (globulins), and alcohol-soluble (prolamins) fractions. To extract water-soluble protein, 1 ml of 25 mM Tris was added to the ground sample, which was then vortexed and centrifuged for 15 minutes at 13,000 rpm. The supernatant was collected and quantified after centrifugation using the Bradford Assay (Thermo Scientific, California). Subsequently, a protein extraction solution containing 10 mM Tris-HCl (pH 7.5-8), 1 M NaCl, 0.1% (w/v) DTT, and 10 mM EDTA was used to extract salt-soluble protein. 1 ml of the extraction solution was added to the remaining solid. The sample was placed in an orbital shaker for 2 hours. Afterward, it was centrifuged for 15 minutes at 15,000 g at 4°C, and the supernatant was again quantified using the Bradford Assay. Finally, the remaining pellet was treated with 1 ml of 70% ethanol to extract alcohol-soluble proteins (prolamins), incubated for 1 hour with shaking, and centrifuged. All protein fractions were quantified separately using the Bradford Assay (Thermo Scientific, California) and combined to estimate total storage proteins.

### 2.5 Candidate gene analysis for flowering time and seed shattering

Variation in flowering time and seed shattering are some of the major bottlenecks for hemp cultivation, with economic challenges. To address these issues, we investigated candidate genes such as *CsCENLP* and *CsDrrp2*, which may be involved in developmental processes related to flowering and cell wall composition, factors that contribute to seed pod dehiscence and dispersal. Understanding the molecular basis of these traits is crucial for developing improved cultivars with uniform agronomic performance and reduced seed shattering.

#### 2.5.1 Selection of candidate genes

Two candidate genes were selected in *Cannabis sativa* based on homology to the *Glycine max* genome from the Pink Pepper Cannabis genome. A predicted gene, annotated as disease resistance response protein 206 (*CsDrrp2*), showed the highest sequence identity to the soybean *GmPdh1* gene involved in pod shattering. Similarly, *CEN-like protein 2* (*CsCENLP*), located on Chromosome 2 (LOC115720824), was identified as a homolog of the *soybean terminal flower 1b* (*GmTFL1b* or *Dt1*) gene, which regulates flowering.

Gene-specific primers were designed for both candidate genes. The forward primer sequence for *CsDrrp2* was CCCATTACACTCGACAACAATC, and the reverse primer was CCACCAACCGAAACATC. Similarly, the forward primer sequence for *CsCENLP* was GTGATAGGAGATGTGGTTGATGT, and the reverse primer was TGCTCCCTCAAATAAGGATCAC. The amplicon size for *CsDrrp2* is 200 bp, while the male flower for *CsCENLP* is a 334 bp band, and the female flower is two bands of 334 and 380 bp.

#### 2.5.2 Nucleic acid extraction

Plant tissues were collected at different developmental stages from the Carver field experiment and flash frozen. Total RNA was extracted using the TRIzol method according to the manufacturer’s instructions (Thermo Fisher Scientific, California). Genomic DNA was extracted using the modified CTAB method (Aboul-Maaty and Oraby, 2019). RNA and DNA concentrations and quality were quantified using a NanoDrop Lite spectrophotometer (Thermo Fisher Scientific, California).

#### 2.5.3 PCR

Polymerase chain reaction (PCR) amplification was performed using genomic DNA templates. The thermal profile was as follows: initial denaturation at 94°C for 5 minutes, followed by 35 cycles of denaturation at 94 °C for 30 seconds, annealing at 52 °C (*CsDrrp2*) and 47 °C (*CsCENLP*) for 30 seconds, and extension at 72 °C for 1 minute, with a final extension at 72 °C for 7 minutes. The amplicons were visualized on 1.5% agarose gels.

#### 2.5.4 qRT-PCR analysis

First-strand cDNA synthesis was performed using the Thermo Fisher manufacturer’s protocol. Quantitative real-time PCR (qRT-PCR) was conducted in a 15 µL reaction containing 1.5 µL of synthesized cDNA, 7.5 µL of SYBR Green Master Mix, 1.5 µL of a mixed 2 µM primer solution (forward and reverse primers for *CsDrrp2* and *CsCENLP*), and 4.5 µL of nuclease-free water (Biorad MJ mini, California). The thermal cycling conditions were as follows: initial denaturation at 95 °C for 3 minutes, followed by 34 cycles of denaturation at 95 °C for 30 seconds, annealing at 52 °C (*CsDrrp2*) and 47 °C (*CsCENLP*) for 30 seconds, and extension at 72 °C for 30 seconds, with a final extension at 72 °C for 7 minutes.

Relative expression levels were calculated using the 2^^−ΔΔCt^ method. The α-tubulin was used as the internal reference for normalization (Deguchi et al., 2021). Each reaction was performed in three biological replicates.

### 2.6 Data analysis

Statistical analysis was performed using RStudio version 4.3.2. Data from the 2022, 2023, and 2024 seasons were examined separately using one-way analysis of variance (ANOVA). Differences between means for each year were separated using Tukey’s Honest Significance Difference (HSD) test. A probability level of *P< 0.05* was considered significant for F-statistic values and mean comparisons. Graphing was done using the ggplot command from the ggplot2 package in R (R Development Core Team, 2020). Only for the varieties that were grown in all three years, treatment x season interaction was performed using two-way ANOVA in R Studio.

## 3 Results

### 3.1 Germination and emergence percentage

There was significant variation in germination percentage among the varieties in the greenhouse conditions (P< 0.001). Carmenecta had the highest germination percentage at 68%, followed by BVL3 and Gravity. On the other hand, Vega had the lowest germination percentage at 26.83% (**Table 5**).

**Table 5:** Comparison of agronomic traits in industrial hemp varieties.

Variety	Germination (%)	Field emergence (%)	Plant population (plants per m^2)^	Seed shattering (0-5)	Weed intensity (0-5)
Altair	36^cdef^	66^ab^	136^abc^	5^a^	3.5^ab^
Bialobrzeskie	46.65^bcdef^	80^a^	180^a^	4.75^a^	3^b^
BVL1	35.39^cdef^	73^ab^	102^abcd^	4^bc^	2.83^b^
BVL2	32.5def	73^ab^	80^abcd^	3.75^c^	2.83^b^
BVL3	68^ab^	71.5^ab^	65^bcd^	3^de^	2.75^b^
Carmenecta	68^ab^	71.5^ab^	100^abcd^	3.5^cd^	2.08^bc^
Felina	28.54^ef^	71.5^ab^	128^abc^	5^a^	3.78^ab^
Ferimon	30.5^ef^	77^ab^	96^abcd^	5^a^	4.95^a^
Fibror 79	60^bc^	82.5^ab^	131^abc^	4.5^ab^	3.85^ab^
Futura 83	63.5^ab^	77^ab^	167^ab^	3.5^cd^	2.08^bc^
Gravity	63.75^ab^	93.5^a^	128^abc^	3.5^cd^	3.3^ab^
Henola	34.5^def^	82.5^ab^	170^ab^	5^a^	3.25^ab^
Jinma	88.4^a^	77^ab^	86^abcd^	2.75^e^	0.85^c^
Orion	57.25^bcd^	93.5^a^	152^ab^	5^a^	3.85^ab^
Santhica	52.5^bcde^	88^a^	189^a^	5^a^	3.85^ab^
Uso 31	28.5^ef^	77^ab^	107^abcd^	5^a^	4.95^a^
Vega	26.83^f^	77^ab^	37^cd^	5^a^	3.78^ab^
F Value	15.90	0.94	4.64	57.48	6.20
P Value	<0.001	>0.05	<0.001	<0.001	<0.001
Level of significance	***	*ns*	***	***	***

ns, non-significant.

*Significant at p ≤ 0.05.

**Significant at p ≤ 0.01.

***Significant at p ≤ 0.001.

Values followed by different lowercase letters within a row are significantly different at P< 0.05 (Tukey’s HSD test). Jinma, a fiber variety, was used as a check variety for comparison.

However, under field conditions, all the varieties displayed emergence percentages ranging from 66% to 93.5% (**Table 5**). The variation among the genotypes at the field level was not statistically significant (P > 0.05). Gravity and Orion demonstrated the highest emergence percentage of 93.6%. In contrast, Altair had the lowest emergence percentage (66%), followed by Felina (70%) (**Table 5**).

### 3.2 Growth pattern and final plant height

**Figure 1:**
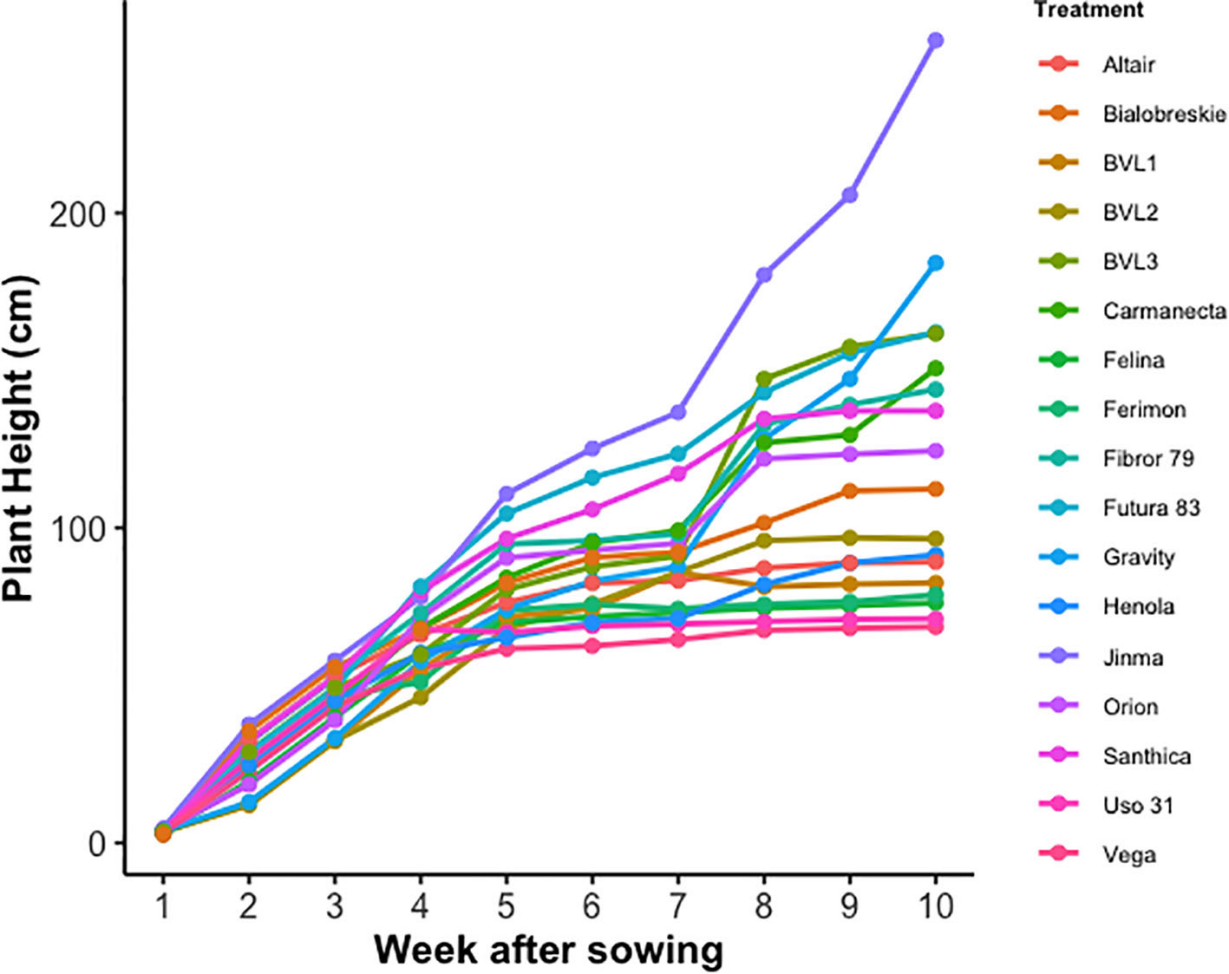
Time series measurement of plant height among fiber, grain, and dual-type industrial hemp varieties. The plant height within each week is the mean of the two replications studied.

The growth pattern from the emergence until harvesting among the different hemp varieties is illustrated in **Figure 1**. Grain type varieties continued growing till 5^th^ week of sowing, after which they transitioned to the flowering stage from the vegetative stage. After the flowering stage, grain-type cultivars Vega, Felina, BVL1, BVL2, Ferimon, Uso-31, and Altair showed minimal growth. Unlike grain-type varieties, dual-type and fiber-type varieties continued growing until the 10^th^ week, providing evidence of longer maturation time (**Figure 1**).

Plants reach the final plant height (FPH) when they cease to grow vertically. FPH is a key plant performance trait that is correlated with plant biomass, lodging, and yield. Furthermore, it is essential for compatibility with mechanized harvesting. The study demonstrated the varietal effect on plant height among different hemp varieties (P value<0.001). The detailed plant height can be found in **Table 1**. BVL3 had the highest plant height, 164.59 cm, among the dual-type varieties tested for the year 2023. Similarly, Altair had the highest plant height of 91.44 cm among the grain-type varieties for the year 2023. On the other hand, Vega (68.07 cm) had the shortest plant height for the same year (**Table 1**).

### 3.3 Plant standing and plant population

For the planting seasons 2022/2023/2024, Futura 83 displayed the highest plant standing, indicating its robust field performance (**Table 1**). On the contrary, grain-type varieties, namely Ferimon, Uso-31, and Vega, displayed the lowest plant standing scores in all these years. While the plant standing provides some insight into the overall qualitative traits of the plants, future studies should incorporate more physiological measurements, such as chlorophyll content and photosynthetic rate, for improved precision. Based on the number of plants per square meter, Santhica (190 plants/m^2^) and Bialobrzeskie (180 plants/m^2^) had the highest plant population (P< 0.01) (**Table 5**).

### 3.4 Weed intensity

All the tested varieties displayed some degree of weed infestation. The dual-type varieties, such as Futura 83 and Carmenecta, had lower weed infestations per plot (weed infestation scores of 2.08 and 2.5) (**Table 5**). This still means weeds covered more than 45% of the plot, a considerable weed pressure. Conversely, the grain varieties were highly susceptible to weed infestation, with Ferimon, Uso-31, and Vega displaying the highest weed infestation. Weeds covered more than 80% of the plots grown with grain-type varieties. The major weed we found was cocklebur (*Xanthium strumarium*) and other weeds we found were Goose grass (*Eleusine indica* (L.) crab grass (*Digitaria sanguinalis* (L.) Scop and other monocots.

### 3.5 Stem diameter

We found significant stem diameter variation across industrial hemp varieties in three years (P value<0.001). Jinma (17.63 mm) had the largest stem diameter among the tested varieties in all three years. Among dual-type and grain-type, BVL3 had the largest stem diameter of 10.66 mm for the planting season 2023. In contrast, Henola, a grain-type variety, had the smallest stem diameter, 4.42 mm (**Table 1**). Like plant height, a discernible pattern was observed in the stem diameter across different hemp types. Fiber variety exhibited the largest diameters, followed by dual-type and grain-type varieties.

### 3.6 Stomatal conductance

**Figure 2:**
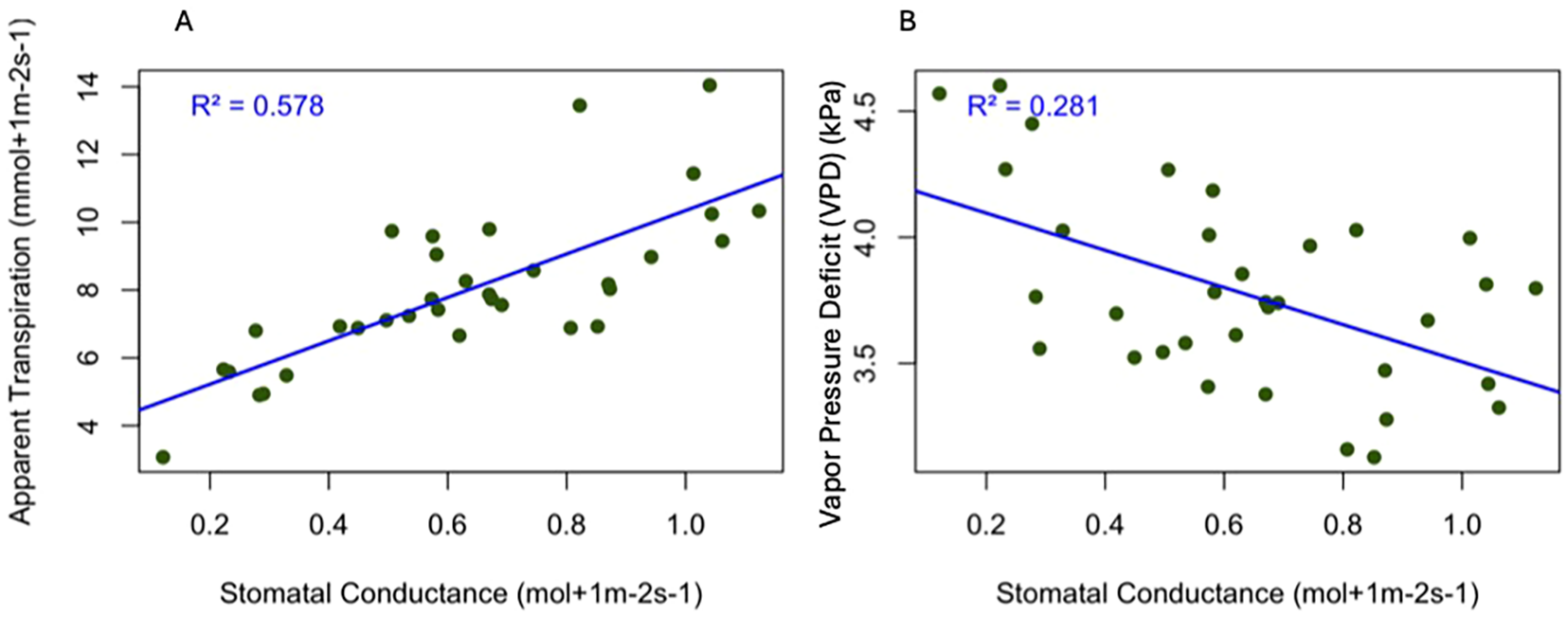
Relationship between stomatal conductance and physiological traits in the evaluated varieties in the hemp plants during the maturation stage. The blue line represents the regression line with data points in green **(A)** gs vs apparent transpiration (R² = 0.578, p< 0.001) **(B)** gs vs vapor pressure deficit (R^2^ = 0.281, p<0.05).

We observed that stomatal conductance (gs) increased with increasing apparent transpiration (**Figure 2A**). A positive linear relationship was observed between stomatal conductance and apparent transpiration rate, with an R² value of 0.57 (P< 0.001), indicating 57% of the variation in transpiration is explained by changes in stomatal conductance. Our result aligns with physiological expectations: higher stomatal conductance leads to more water loss through transpiration. Additionally, we found that the stomatal conductance decreased with increasing vapor pressure deficit (**Figure 2B**). These two variables had a weak relationship (R² = 0.28), meaning only 28 percent of the variation in the stomatal movement in the plants is due to Vapor Pressure deficit (VPD) (P value<0.05).

### 3.7 Flowering and maturity traits

Seeds are considered mature when their moisture content reaches between 18% and 20%. We found that most of the grain varieties flower and mature earlier than dual-type and fiber-type varieties under the photoperiodic conditions (**Table 3**). Additionally, male plants flowered 15 to 20 days earlier than female plants (**Table 3**). Ideally, industrial hemp should be harvested when the seed moisture content is 20-30% for optimal yield. Most of the grain-type varieties matured in 60 to 75 days (**Table 3**). However, two grain type varieties originated from the USA had an intermediate flowering (60–65 days) and maturation time (115–120 days). Likewise, dual-type varieties took 115 to 120 days to mature (**Table 3**). Varieties such as Carmenecta, Gravity, BVL1, and BVL2 had a higher ratio of male plants; nevertheless, they had consistent good yield (**Table 3** and **Table 6**).

**Table 6:** Three-year comparison of grain yield among the hemp varieties.

Variety	Grain yield (kg/ha)	Grain yield (kg/ha)	Grain yield (kg/ha)
	2022	2023	2024
Futura 83	2434.59^a^	2793.20^a^	2581.24^a^
Henola	NA	1881.95^b^	648.14^d^
BVL3	NA	1800.76^bc^	2079.95^ab^
Felina	662.91^bc^	1497.86^bcd^	NA
Carmenecta	1353.68^b^	1471.57^bcd^	2030.85^ab^
BVL2	NA	1312.16^bcde^	NA
Fibror 79	NA	1204.94^cde^	830.95^cd^
GR4	NA	1202.84^cde^	NA
Santhica	NA	1086.62^de^	NA
BVL1	NA	1051.59^def^	506.88^abc^
Bialobrzeskie	619.65^bc^	980.616^def^	589.22^d^
Orion	1004.71^bc^	883.70^defg^	NA
Altair	NA	830.25^efg^	NA
Vega	1250.46^b^	732.12^efg^	1289.18^bcd^
Ferimon	240.41^c^	445.42^fg^	NA
Uso 31	939.77^bc^	329.14^g^	NA
BVL4	NA	NA	1716.90^abc^
BVL5	NA	NA	1084.77^bcd^
F value	10.00	29.14	10.48
P value	<0.001	<0.001	<0.001
Significance	***	***	***

ns, non-significant.

*Significant at p ≤ 0.05.

**Significant at p ≤ 0.01.

***Significant at p ≤ 0.001.

Values followed by different lowercase letters within a row are significantly different at P< 0.001 (Tukey’s HSD test). “NA” varieties shown in the table legend represent those varieties that were not planted in that year.

### 3.8 Biomass

There was a statistically significant difference in the whole plant, above-ground, and below-ground biomass among the varieties studied (P value< 0.001). Based on this study, Futura 83 had the highest biomass, 111523.51 kg/ha, followed by Carmenecta. On the other hand, Vega had the lowest biomass (**Table 7**). Likewise, aboveground biomass, which provides the biomass for actual use, also differed significantly among the evaluated varieties (P value<0.001), with the variety Futura 83 posing the highest above-ground biomass. Likewise, below-ground biomass was highest on the dual type variety Carmenecta, comparable statistically with the check fiber type variety.

**Table 7:** Comparison of biomass among industrial hemp varieties for the planting season 2023.

Variety	Whole plant biomass (kg/ha)	Above-ground biomass (kg/ha)	Below-ground biomass (kg/ha)
Altair	14960.10^bc^	10984.26^bc^	2051.43^bc^
Bialobrzeskie	25636.57^bc^	22869.34^bc^	2137.72^bc^
BVl1	8596.62^c^	5244.70^c^	102.03^c^
BVL2	6454.14^c^	5019.07^c^	560.20^c^
BVL3	18504.27^c^	23677.93^abc^	1814.75^c^
Carmenecta	36518.01^ab^	49959.74^ab^	9369.70^ab^
Felina	10781.91^c^	5692.21^c^	525.33^c^
Ferimon	3543.23^c^	5126.26^c^	319.51^c^
Fibror 79	34248.34^abc^	15316.18^bc^	3289.29^bc^
Futura 83	111523.51^a^	51971.05^a^	9217.54^ab^
Gravity	29687.85^abc^	18562.14^bc^	5623.91^abc^
Henola	11237.35^c^	5508.16^c^	3322.29^bc^
Jinma	59647.11^ab^	38544.50^ab^	11692.19^a^
Orion	32651.93^abc^	26590.40^abc^	7370.96^abc^
Santhica	23604.27^bc^	23207.63^abc^	5364.54^abc^
Uso 31	6613.83^c^	4220.63^c^	226.81^c^
Vega	1222.54^c^	1228.14^c^	160.81^c^
F value	6.32	13.57	8.45
P value	<0.001	<0.001	<0.001
Statistical significance	***	***	***

ns, non-significant.

*Significant at p ≤ 0.05.

**Significant at *p* ≤ 0.01.

***Significant at *p* ≤ 0.001.

Values followed by different lowercase letters within a row are significantly different at P< 0.05 (Tukey’s HSD test). Jinma, a fiber variety, was used as a check for comparison.

We performed linear regression analyses on whole plant biomass against other key traits influencing this characteristic. We found that stem diameter, final plant height, and below-ground biomass strongly correlated with the whole plant biomass, with R-squared values greater than 70% (**Supplementary Document 1-File 9**).

### 3.9 Grain yield

We conducted a multi-year varietal trial to screen suitable varieties for the Midwest production regions and to identify parental lines for breeding purposes. 2022 was the starting year for our research, and given the limited seeds available, we screened nine varieties. During the 2022 planting season, the highest grain yield among eight evaluated varieties was from Futura 83, a French-origin variety, with a grain yield of 2434 kg/ha. The field trial continued in 2023 with a total of 16 varieties, including Futura 83 and other good-performing varieties. During the 2023 planting season, the Futura 83 again achieved the highest grain yield of 2,793 kg/ha. There was an increase in the grain yield compared to 2023. Similarly, in 2024, among the 10 varieties, Futura 83 consistently had a higher grain yield compared to other varieties, but it was lower than in 2023, with a grain yield of 2581 kg/ha (**Table 6**). Despite different climatic conditions in multiple years (**Supplementary Materials Document 1-file 2**), Futura 83 had a consistent grain yield on average of 2602 kg/ha for three consecutive years, suggesting stable grain production. In contrast, grain varieties such as USO-31, Ferimon, and Bialobrzeskie had lower grain yield in all the planting years (**Table 6**).

In addition to Futura 83, BVL3, a USA-origin variety, showed good grain yield in 2023 and 2024. The grain yield for 2023 and 2024 was 1800 kg/ha and 2079 kg/ha, respectively. Additionally, it has a high plant biomass (22.26 g/plant). The percentage of male flowers in Futura 83 is very low, the male percentage in BVL3 is 45%.

### 3.10 Seed compositional trait

#### 3.10.1 Crude protein and total seed storage protein

**Figure 3:**
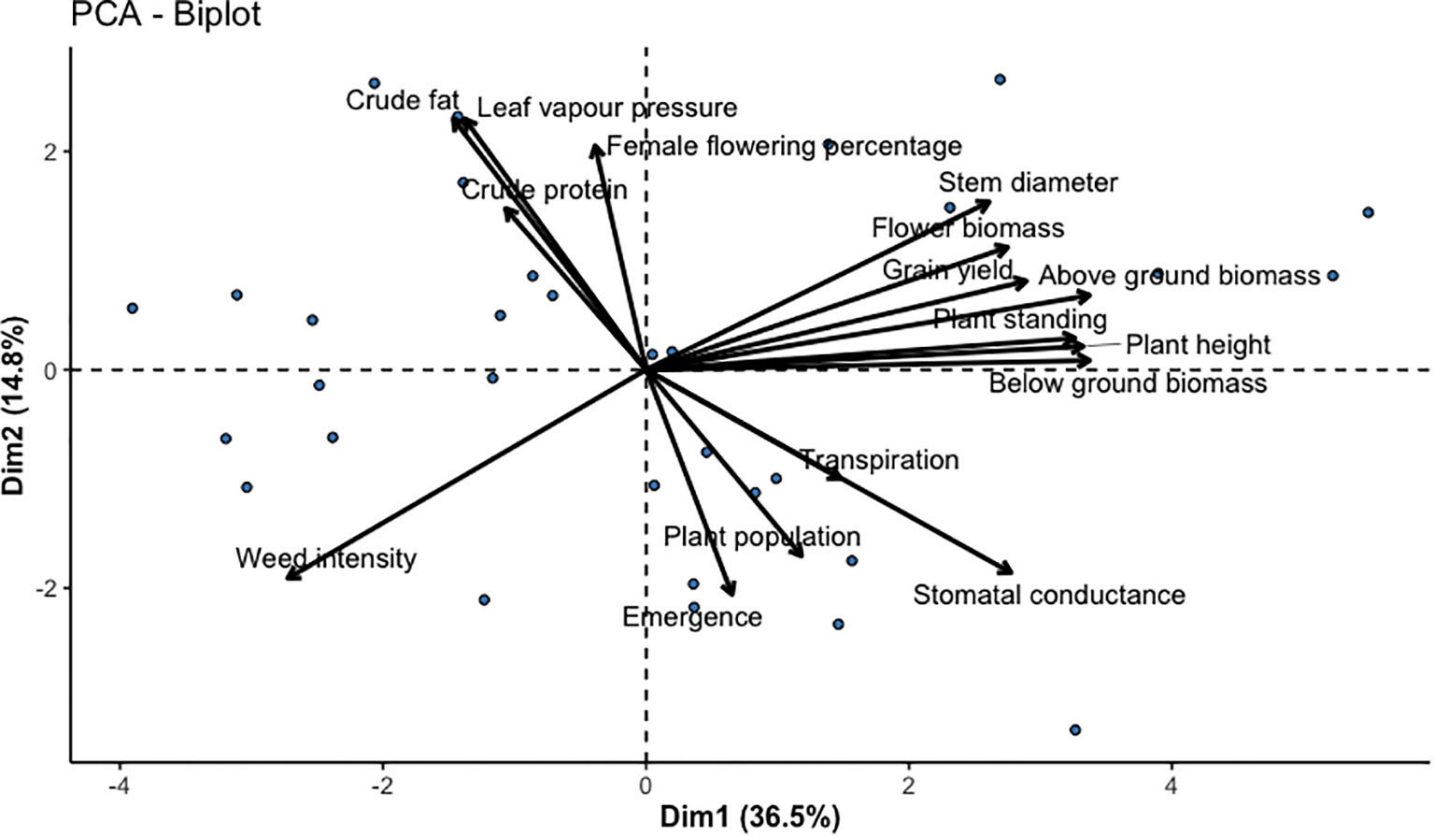
PCA biplot of 16 grain and dual-type industrial hemp varieties. The principal component analysis (PCA) of yield and plant performance traits.

The statistical test revealed a significant difference in seed proteins among the evaluated varieties (P-value<0.001). We found that the crude protein ranged from 23% to 27% on a dry seed basis. Ferimon (27.57%) and Futura 83 (26.48%) had the highest crude protein content. Similarly, the total seed storage protein ranged from 26.81% to 30.35% per gram of dry seeds. The water-soluble protein concentration ranged from 12.98% to 17.11% per gram of dry seed. Among the varieties studied, Futura 83 had the highest soluble protein content of 17.12%, followed by Ferimon, 16.89% (**Table 8**). Among the seed compositional traits, crude protein had relatively less variability among the tested varieties compared to crude fat and ash content (**Table 8** and **Figure 3**).

**Table 8:** Comparison of key seed compositional traits in industrial hemp varieties.

Variety	Total seed storage protein (%)	Crude protein (%)	Soluble protein (%)	Crude fat (%)	Mean crude fiber (%)	Mean ash (%)
Altair	27.96^ab^	24.54^bcd^	14.14^abc^	30.54^bcd^	22.12^abc^	4.57^e^
Bialobrzeskie	27.43^ab^	24.53^bcd^	13.63^abc^	32.24^ab^	20.79^c^	5.27^b^
BVL1	29.04^ab^	25.69^ab^	15.25^abc^	28.8^def^	23.10^ab^	4.38^fg^
BVL2	26.94^b^	24.47^bcd^	13.60^abc^	28.74^def^	24.28^a^	5.14^c^
BVL3	26.94^b^	24.52^bcd^	13.45^abc^	28.26^ef^	24.13^a^	4.49^ef^
Carmenecta	27.98^ab^	24.77^bcd^	14.18^abc^	29.88^cde^	21.62^bc^	5.58^a^
Felina	28.58^ab^	25.90^ab^	14.78^abc^	31.39^abc^	20.49^cd^	4.23^h^
Ferimon	29.83^ab^	27.57^a^	16.89^ab^	30.93^abc^	20.11^cd^	4.46^ef^
Fibror 79	29.91^ab^	25.62^abc^	16.11^abc^	28.09^ef^	20.39^cd^	5.22^bc^
Futura 83	30.83^a^	26.48^ab^	17.11^a^	32.86^a^	18.36^d^	5.52^a^
GR4	28.97^ab^	23.34^cd^	15.68^abc^	22.88^g^	23.06^ab^	5.53^a^
Henola	27.93^ab^	23.30^d^	14.13^abc^	29.56^cdef^	24.32^a^	4.19^h^
Orion	27.04^b^	24.96^bcd^	13.24^bc^	28.10^ef^	21.32^bc^	4.87^d^
Santhica	26.81^b^	25.35^abcd^	12.98^c^	29.53^cdef^	21.72^bc^	4.27^gh^
US 30	28.69^ab^	25.63^abc^	14.89^abc^	30.35^bcd^	21.23^bc^	4.79^d^
Vega	28.96^ab^	24.75^bcd^	15.36^abc^	21.66g	23.47^ab^	5.36^b^
F value	2.98	5.48	3.0153	54.03	15.12	339.65
P value	<0.001	<0.001	<0.001	<0.001	<0.001	<0.001
Significance	***	***	***	***	***	***

ns, non-significant.

*Significant at p ≤ 0.05.

**Significant at p ≤ 0.01.

***Significant at p ≤ 0.001.

Values followed by different lowercase letters within a row are significantly different at P< 0.001 (Tukey’s HSD test).

#### 3.10.2 Crude fat

Likewise, crude fat content significantly varied among the varieties (P<0.001). Crude fat in the evaluated varieties ranged from 21.66% to 32.86%. The highest crude fat content was found in Futura 83 (32.86%) and Bialobrzeskie (32.24%), which are primarily grown for grain yield, and the lowest crude fat content was seen in Vega (21.66%) (**Table 8**). Compared to protein, which exhibited moderate variability, crude fat showed greater phenotypic variability (**Table 8** and **Figure 3**).

#### 3.10.3 Crude fiber and ash

Similarly, there was also a significant variation in crude fiber and ash content across varieties (P< 0.01). The highest crude fiber content was found in BVL2 (24.28%), Henola (24.32%), and BVL3 (24.13%) (**Table 8**). Ash is made up of inorganic compounds and determines the total mineral content of a sample. Our result suggests the ash content to be highest in Carmenecta (5.58%) and GR4 (5.53%) (**Table 8**), which are both dual-type varieties.

#### 3.10.4 Principal component analysis of the evaluated agronomic traits

Principal component analysis (PCA) was employed to evaluate the multivariate structure of 4 yield-related and 11 plant performance traits across diverse hemp varieties. PC1, explaining 38.8% of the variation, was predominantly influenced by above-ground biomass, whole plant biomass, below-ground biomass, and harvestable plant height, indicating that biomass accumulation and plant height are key drivers of inter-varietal differences (**Figure 3**). PC2, accounting for 14.6%, was shaped by plant population, emergence percentage, and stomatal conductance, suggesting that early vigor and physiological efficiency also play essential roles in varietal performance. The PCA biplot (**Figure 3**) illustrated the strength and direction of trait contributions, with closely grouped vectors indicating positive correlations, particularly among biomass-related traits. In contrast, opposing vectors highlighted negative trait associations, such as between emergence and weed percentage. PCA-reduced data were subjected to cluster analysis to explore phenotypic variation further, resulting in three distinct varietal groups visualized with convex hulls (**Supplementary Document 1-File 18**). Overall, this combined PCA provided a robust framework for selecting hemp genotypes with desirable agronomic traits and informs targeted breeding strategies for biomass, plant vigor, or physiological resilience.

#### 3.10.5 Pearson’s correlation among agronomic traits

**Figure 4:**
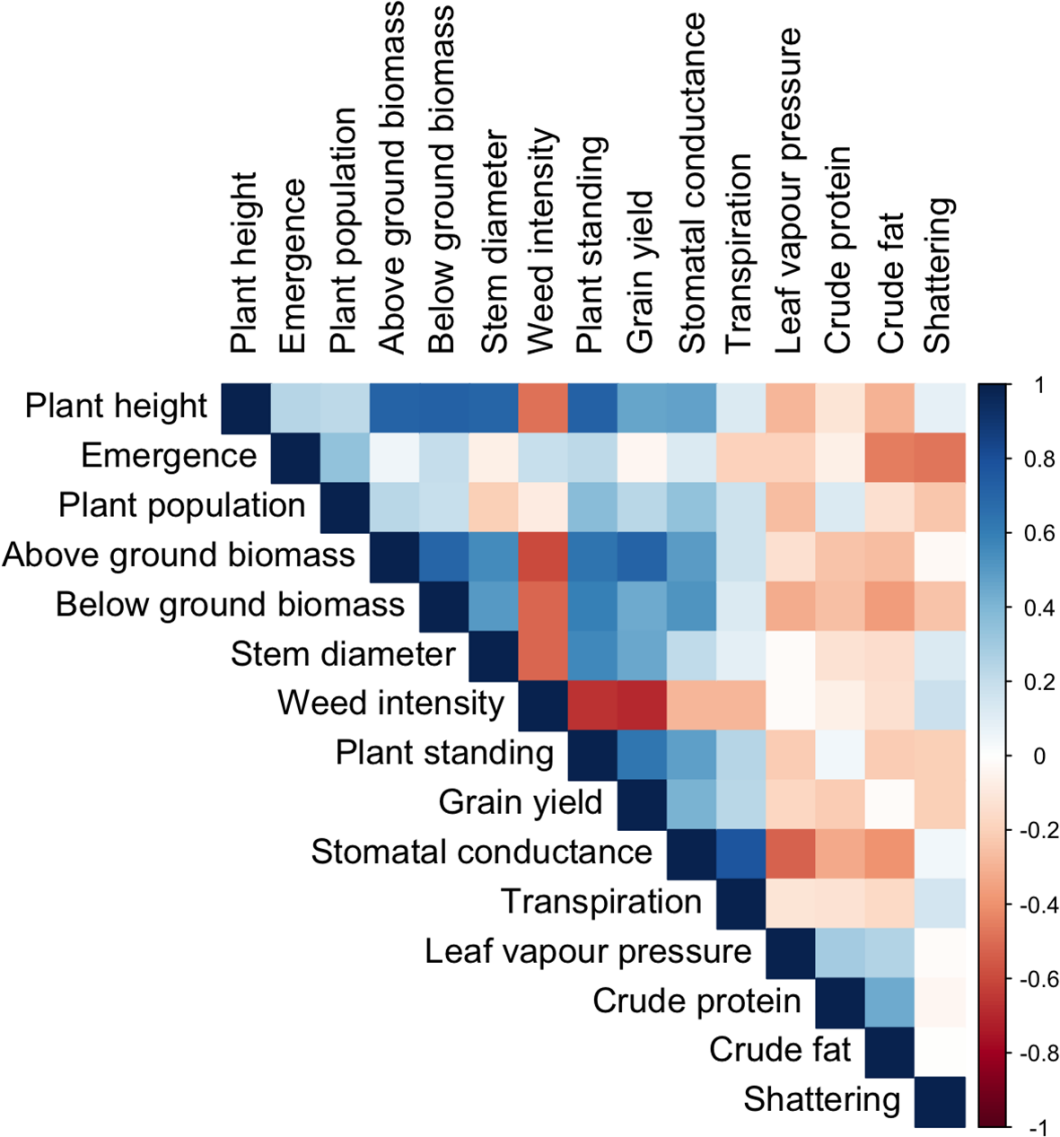
Pearson’s correlation matrix (r) shows an association between key physiological, agronomic, and seed compositional traits. ns p>0.05; *<0.05,**<0.01; and ***<0.001.

Grain yield, aboveground biomass, crude protein, and crude fat are economically essential traits in industrial hemp. In our study, grain yield was highly correlated with aboveground biomass (r = 0.72) and plant standing ability (r = 0.63). It also showed a moderate but significant positive correlation with plant height (r = 0.47) and below-ground biomass (r = 0.45). These findings suggest vigorous plants with greater aboveground biomass produce higher grain yields (**Figure 4**).

Aboveground biomass was also strongly correlated with plant height (r = 0.72), below-ground biomass (r = 0.71), and stem diameter (r = 0.56). In addition, stomatal conductance was moderately correlated with plant height, aboveground biomass, below-ground biomass, and plant standing. It also exhibited a moderate positive correlation with grain yield, indicating that stomatal function may regulate plant productivity and structural integrity under field conditions (**Figure 4**).

In contrast, crude protein and fat content did not correlate significantly with the measured agronomic traits, suggesting that distinct physiological or genetic mechanisms may govern these seed quality attributes (**Figure 4**). All the detailed correlation values can be found in the **Figure 4** Correlation analysis result.

### 3.11 Linking agronomic traits to genetic regulation: Expression of *CsDrrp2 and CsCENLP*

**Figure 5:**
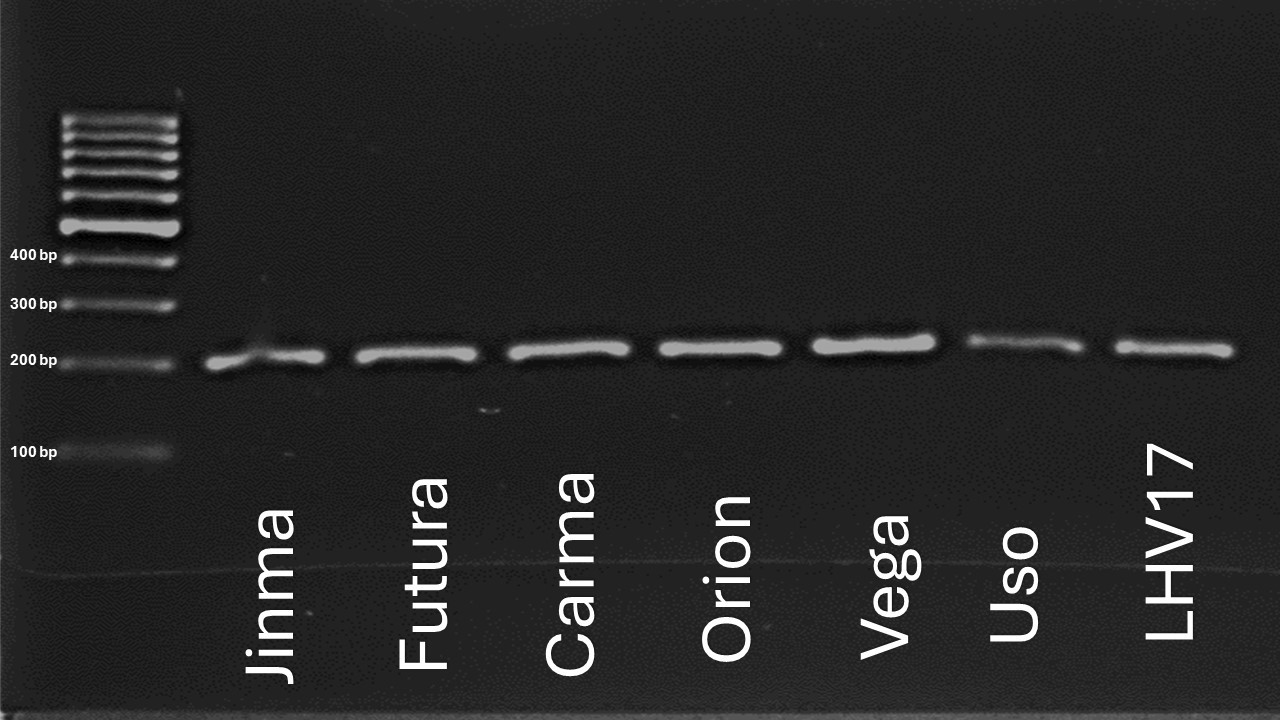
Screening of the CsDrrp2 gene across selected industrial hemp varities.

We found that the homolog of the *GmPdh1* gene (*CsDrrp2*) is present in the selected industrial hemp varieties, including the wild-type hemp accession (LHV17) (**Figure 5**). To further understand its gene expression pattern, RT-PCR was conducted to examine the *GmPdh1* homolog gene (*CsDrrp2*) gene expression among various hemp tissues in the early and late reproductive stages, Futura 83.

The seed development in hemp undergoes the following stages (The figure is demonstrated in **Supplementary Document 1 File 8**).

Flowering stage (R1): The stage when the first female flower is seen.

Early seed development stage (R2): The stage when the seed begins developing from the female flower. No seed shattering was seen in this stage.

Early seed filling stage (R3): The stage when the seeds are developed but still soft and milky when crushed with a finger. There was no shattering in this stage.

Later seed filling stage (R4): The stage when the seeds become hard and begin shattering, detaching from the mother plant, and spreading away from the middle lamella. The shattering was mild in the beginning and severe in the later stage.

Mature seeds (R5): The stage when the seed has entirely shattered and lies on the ground.

We observed two dehiscence zones that induced shattering. One was in the bracts that contain each seed (**Supplementary Document 1 File 8**). Another zone was in the chalaza placental region, where the seed is physically attached to the mother plant. In most cases, shattering is because of the dehiscence zone I in the bracts (**Supplementary Document 1 File 8**).

**Figure 6:**
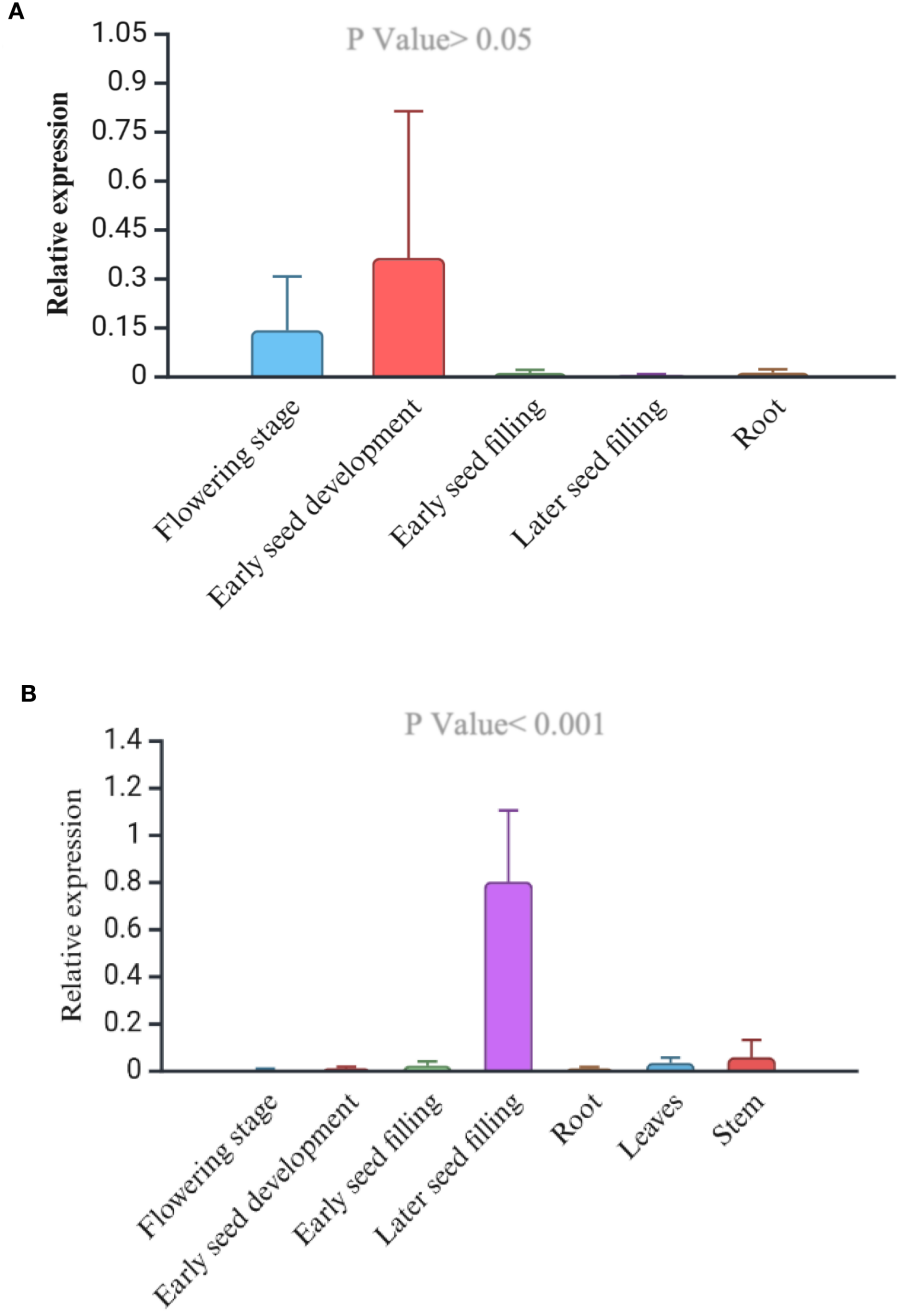
Relative expression of the *CsDrrp2* gene (*GmPdh1* homolog) in the successive plant development **(A)**, Wild-type industrial hemp variety. The tissue-specific expression is not significant (P value>0.05, F value 0.51) **(B)**. Futura 83, a cultivated dual-type hemp variety (P value<0.001, F value 19.226).

**Figure 7:**
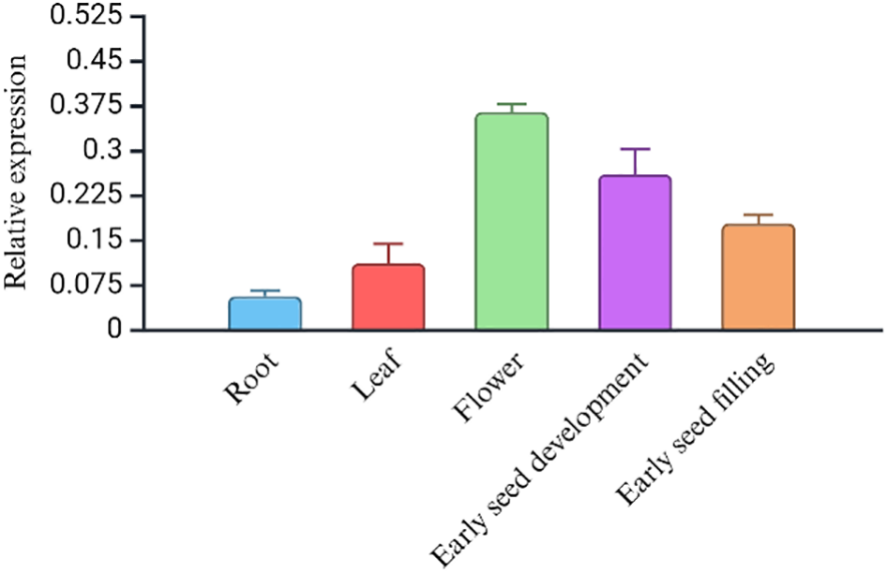
The gene expression profile (RT-PCR) of the *CsCENLP* in different tissues in Futura 83. (P value<0.001, F value 181.89).

Phenotypically, the early flowering variety, USO-31, shatters more than the later-flowering fiber variety Jinma and Futura 83 (**Table 5**). Our field study suggests that the varieties should have 65–70 days for maximum grain yield and less shattering (**Table 5**). We studied the expression levels of CsDrrp2 at different stages of seed development in Futura 83 and LHV 17. There was a higher expression of this gene in the reproductive tissues than vegetative tissues in both Futura 83 and LHV 17. However, there was not statistical significance of differential expression in LHV-17 (**Figures 6A, B**). Similarly, we found *CsCENLP* to be expressed in all the tissues, particularly more in flowers and seeds than root (**Figure 7B**).

## 4 Discussion

Most of the varieties we screened originated from Europe and Canada (**Table 2**). Missouri has distinct climatic differences compared to these regions. For instance, Missouri experiences hot and humid summers, whereas countries such as France, where varieties like Futura 83, Orion, and Fibror 79 were bred have milder summer conditions (NOAA, 2023). Missouri’s summer temperatures are also generally higher than those in Italy, Ukraine, Poland, and Canada (Ferfuia et al., 2021).

Additionally, Missouri’s lower latitude results in shorter summer photoperiods, which can trigger premature flowering in photoperiod-sensitive crops like hemp. European cultivars bred in France, Italy, and Poland typically come from more stable climates, and the uneven rainfall patterns in Missouri may negatively impact their performance (Campiglia et al., 2017). Similarly, Canadian varieties are bred for shorter, drier growing seasons and may not be well-suited to Missouri’s hot and humid climate. Although the use of two replications was appropriate for an early-stage screening trial, we acknowledge that it limits the statistical power and robust conclusions, particularly for traits such as yield and biomass influenced by environmental variability. Future experiments will include additional replications and site locations to bolster the varietal performance assessments and to delve deeper into genotype × environment interactions. The subsequent paragraphs describe the plant phenology and yield of the tested varieties in Missouri’s production environment.

### 4.1 Germination and plant growth

We observed all the varieties to have better germination in the field conditions than in the greenhouse conditions. Light irrigation in the field followed by seed sowing might have contributed to better germination in the field. In addition, fertilized soil, on the contrary to just the cocopeat used in the greenhouse, might have favored the germination in the field. However, further study should be conducted for further verification. We found that the different varieties had distinct growth patterns and time frames to transition from one growth phase to another. Grain-type varieties have earlier flowering and maturation times than fiber-type varieties. Like our study, Rahemi et al. (2021) and Ferfuia et al. (2021) also found that grain-type varieties, such as USO 31, had earlier flowering and maturation than fiber-type varieties. These phenological differences among the varieties might be because of the distinctive degree day requirement for each variety and the climatic differences between Missouri and the country where it was bred (Ferfuia et al., 2021). These findings would be critical for regional cultivar selection; short-maturing types could be selected in the Northern part of the United States, and later-maturing types could be selected for the Southern Region.

### 4.2 Planting population

We observed differences in plant population among the tested varieties. This might be due to a combination of factors, such as poor germination, adverse soil conditions, and biological threats like insects and pathogens (Van Roekel and Coulter, 2011). Like our experiment, Rahemi et al. (2021) also reported the grain varieties to have higher plant populations. The higher plant population found in Santhica and Bialobrzeskie v might be because of its smaller size and the intrinsic seed vigor traits that facilitate more germination and seedling emergence (Huang et al., 2019).

### 4.3 Weed suppression potential among varieties

Weed infestation is one of the significant challenges in industrial hemp cultivation, as there are limited herbicides available for industrial use. Our study did not use herbicides, thereby looking for inter-varietal weed suppression. We found that fiber variety, Jinma, contrary to grain and dual types, could suppress weeds efficiently. The fiber variety grew vigorously and formed a faster, denser leaf canopy, depriving weeds of sunlight and nutrients, thereby suppressing their growth. Furthermore, planting time also affected weed suppression. Notably, the earlier planting facilitated enhanced plant growth, outperforming weeds in fiber and dual-type varieties. Our findings were consistent with the similar findings in other cropping systems, which showed that early planting improves crop dominance over the weeds through faster establishment and resource capture (Williams, 2006). Based on our findings, fiber-type hemp varieties are recommended for fields with persistent weed pressure, primarily when herbicide use is restricted. Furthermore, we found an inverse relation of weed percentage with regard to the plant population (**Figures 3, 4**) and increasing seeding rate might potentially reduce the weeds; however, this requires further investigation for confirmation. Our weed intensity is based on a visual estimate and future research should include quantitative parameters such as weed biomass, nutrient competition, and additive findings.

### 4.4 Phenotypic variation in plant height and stem diameter:

Our findings on plant height and stem diameter were similar to those in previous research conducted in other parts of the United States. Notably, all the researchers found the fiber varieties to be the tallest, followed by the dual and grain types (Cornell Hemp Trial Report, 2020; Darby, 2021; Rahemi et al., 2021). Plant height (PH) is primarily influenced by stem elongation because of continuous cell division and the expansion of cells generated by the apical and intercalary meristems. In most plants, the plant height and diameter are polygenic traits (Wang and Li, 2008). In addition to genes, plant height is also regulated by plant hormones, including gibberellin (GA), brassinosteroid (BR), auxin (IAA), cytokinin (CK), and strigolactone (SL) (Tong et al., 2009). However, there has been a limited study conducted on the genetic architecture that determines the plant traits, such as plant height and stem diameter, in cannabis. While our study found the plant height to be associated as a varietal trait, further research should study what particular genetic mechanism and hormonal regulation contribute to the distinctive phenotype observed among the varieties (Petit et al., 2020b).

### 4.5 Grain yield

Grain yield is an important trait for the dual-type industrial hemp varieties (Alonso-Esteban et al., 2022). The grain yield among the tested varieties ranged from 328 kg/ha to 2,796 kg/ha. The average grain yield for the varietal trial was 1,283 kg/ha, which is higher than the national average of 896 kg/ha National Agricultural Statistics Service (2022). In a varietal screening study by Das et al. (2020), grain yields ranged from 27 kg/ha to 2,362 kg/ha, aligning with the range observed in this study. Similarly, Rahemi et al. (2021) reported an average grain yield of 771 kg/ha, which also falls within the range found in this study. As in the present research, *Futura 83* demonstrated superior yield and performance traits in both New York and Vermont, confirming its suitability for cultivation across a wider geographic area (Cornell Hemp Report, 2020; Darby, 2021).

Grain yield is a complex trait. Hemp grain production varies among varieties and is influenced by environmental conditions such as soil type, moisture levels, and agronomic practices (Campbell et al., 2019; Campiglia et al., 2017; Poudel et al., 2020; Rahemi et al., 2021; Stack et al., 2021; Toth et al., 2020). In a study conducted across 14 cultivars in Latvia, the Czech Republic, France, and Italy, the grain yields were primarily determined by variety type and then by flowering time, which is consistent with our findings (Campiglia et al., 2017). We also found that grain yield was strongly influenced by variety and flowering time, as observed by Visković et al. (2024).

The climate study by Ferfuia et al. (2021) found that high temperatures during early grain filling negatively impact seed yield. While we did not study the impact of temperature on the yield, we found that early maturing varieties that had a maturation time of 55–60 days had lower yield. Dual-purpose varieties such as Futura 83 produced high grain and biomass yields, which has intermediate maturation times of 120 days. We observed higher biomass to be related to more grain yield, but this trait also additionally implies to higher fiber strength, assimilable sugars, making it more readily available for biofuel and fiber production (Williams et al., 2025). In addition to varietal effects and flowering time, grain yield is highly influenced by the Nitrogen dosage (Tang et al., 2022; Podder et al., 2024). The national average for grain yield is rising, probably because of the discovery of better agronomic practices, genetics, and screening stable varieties in the USA. However, no single variety has been identified that performs well in all the geographic regions of the United States, suggesting the paramount importance of this study (Williams et al., 2025). However, this study used only two replications per site-year, which may reduce the precision of traits such as flowering time and grain yield, especially given their strong environmental interactions. Despite this limitation, the consistent yield observed across multiple years suggests stable genetics in the Futura 83 variety. Nevertheless, future studies should incorporate more replications and multi-environment trials to allow for more robust statistical inference and to better support breeding decisions. Although our study did not delve deeper into the sexual dimorphism and grain yield, this factor might play an important role in this trait. While excessive male flower production can reduce seed yield in dioecious varieties, monoecious plants may produce inadequate pollination. Additionally, in hemp, monoecious might require allocating much resources to pollen production, reducing the resources available for seed development. On the contrary, in a variety of dioecious plants. In some cases, early male plants may senesce before females reach full reproductive maturity, enabling more effective seed development by females. While our study just explored the varietal effect on the yield, future research should explore how distribution of male and female flowers may impact yield parameters.

### 4.6 Seed protein and oil composition

We studied the phenotypic variation of key seed compositional traits such as total protein, crude protein, crude fat, and crude fiber, which are key components for human food and animal feed. The total protein observed in the hemp was 26-31% per gram of seed, similar to the previously identified range of 25%-30% (Aluko, 2017; Sun et al., 2021; M. Liu et al., 2023). Likewise, the crude protein in industrial hemp was 23-27% per gram of seed on a dry weight basis. Our study was consistent with the research findings by Alonso-Esteban et al. (2022), which found hemp seeds to contain between 25% and 35% crude protein and between 20% and 25% crude oil. We found Ferimon and Futura 83 to have the highest crude protein, with 27% and 26%, respectively. These studies further provide evidence of hemp seeds as a rich source of digestible proteins for humans and animal feed (House et al., 2010; Alonso-Esteban et al., 2022). Furthermore, Edestin could be used as a substitute for blood plasma when plasma is lost (such as in trauma and surgery) (Horska, 2005).

A complex genetic network controls grain filling. *Edestin 1 and Edestin 2* encode for edestin protein, the predominant protein in industrial hemp grain (Deguchi et al., 2020; Cattaneo et al., 2021; Sun et al., 2021). Seed storage protein (SSP) encoding genes are primarily activated during the grain-filling and maturation stages. SSPs accumulate in the embryo or endosperm of the seed, depending on the plant species. The endosperm remains a single-cell layer in dicotyledonous plants such as industrial hemp. At the same time, the embryo cells develop protein storage vacuole (PSV) structures to store SSP (Yang et al., 2022). Although significant development has been made in understanding seed protein and storage reserve accumulation in model crops, little work has been conducted on this topic in industrial hemp (Yang et al., 2022). The cause of the variation in seed proteins among the tested genotypes is unknown. The higher total and crude protein observed in Futura 83 and Ferimon 79 varieties may be attributed to their distinct genetic makeup, the environment favoring this cultivar, or the interaction of both genes and the environment. These high-protein-containing cultivars, such as Futura 83 and Ferimon 79, likely possess traits that enhance the expression of these seed storage encoding genes. Increased expression of these genes could result in higher seed protein content in these varieties. However, further investigation is needed to understand the mechanisms governing protein synthesis in hemp comprehensively.

### 4.7 Genetic and environmental roles in flowering

One major challenge in hemp cultivation is the lack of uniform flowering and seed maturation. Some plants may remain vegetative within a single field while others reach full maturity. To exacerbate shattering, some flower clusters fully mature to seed within a single plant, while others are still in intermediate maturity (**Supplementary Material Document 1 File 8**). One way to fix the seed shattering in hemp is to restore the flowering time and engineer it to flower uniformly. The hemp breeding goal should target an intermediate flowering time to reduce seed shattering. Flowering in hemp is often triggered by signals received by the meristematic tissue related to alterations in environmental factors such as light intensity, daylength, influencing flowering time and intensity in industrial hemp (Downs and Borthwick, 1956; Petit et al., 2020b). The plants flower prematurely if they experience daylight periods shorter than 14 hours (Hall et al., 2012; Cheng et al., 2022).

Thus, understanding the mechanisms governing flowering, sexual orientation, and their relation to seed development and shattering is necessary for hemp breeding and improvement (Toth et al., 2020; Asiamah et al., 2025). Our study found the flowering time was different among the tested varieties. However this trait in hemp is very photoperiodic and future research should include how the light intensity, day length and other environmental factors and study whether thresholds vary among varieties to better determine which traits are most strongly conserved (Hall et al., 2012; Zhang et al., 2025). In the meantime, our study remains valuable for preparedness in cultivation and harvesting. Further study is required to characterize the gene expression among different early, late, and intermediate flowering varieties to better understand the flowering patterns and maturity in hemp varieties, and this will help design molecular breeding strategies for flowering time and maturity traits. Additionally, future research should sequence the sex specific gel bands and compare with the available pangenome for the validation (Lynch et al., 2025). While *CsCENLP* has not been directly linked to flowering and shattering, this gene might regulate the timing and the place in the tissues of lignin deposition, which is a very critical physical phenomenon for shattering. Future transcriptomics research and histological research on *CsCENLP* mutants or overexpression lines could give a better overview of the relationship between *CsCENLP* and seed shattering.

### 4.8 Molecular basis of seed shattering

In dicot plants, such as industrial hemp, seed shattering is caused by the development of a specialized dehiscence or abscission zone along the seed cover. During the seed development stage, cells in this zone differentiate into lignified and separation layers, which later undergo self-degradation, after which the seed cover splits open (Maity et al., 2021). Lignification is a necessary process involved in seed shattering. Alongside lignin, other key components of the plant’s secondary cell wall, such as cellulose and hemicellulose are related to cell wall strength and integrity, directly affecting pod dehiscence. Higher cellulose, hemicellulose, and lignin contents in pods are strongly associated with dehiscence (Hofhuis et al., 2016).

In soybean, *GmPdh1* encodes a dirigent (DIR)-like protein. It enhances pod dehiscence by increasing the torsion of dried pod walls, generating a driving force for pod splitting under low humidity conditions (Funatsuki et al., 2014). The homolog of the *GmPdh1* gene is the *CsDrrp2*, which we hypothesized to be associated with shattering. While the expression of the Cs*Drrp2* in the later seed development stage might associate with seed shattering, however, the exact role of this gene is unknown and we cannot provide its function based on the current result. It is unknown whether it causes lignin deposition in the dehiscence layer or cellulose deposition. The homolog of *CsDrrp2* promotes lignification in the inner sclerenchyma of pod walls, the site of thick secondary cell wall formation (Funatsuki et al., 2014; Marsh et al., 2023). This gene may play a similar role in *Cannabis sativa* by promoting lignification in the inner sclerenchyma of the seed coat, as observed in legumes. Such activity could enhance the differential lignin deposition between the inner and outer layers of the seed coat. During consecutive drying cycles, these differences may generate dehiscence forces that lead to seed shattering. The future direction should be RNA sequencing of the highly shattering grain type varieties and the less shattering ones based on our screened varieties for seed development and shattering genes. Furthermore, additional seed shattering genes such as SHAT-5, *SHP1* and *SHP2* that are found to be associated with shattering should also be studied for further exploring the mechanism of shattering (Maity et al., 2021). Shattering occurs as a torsion in the bracts surrounding the seeds, pulling both the seed covers and exposing the seeds. Although our experiment included the bracts surrounding the seeds, further research should focus on identifying the specific tissue and the region where this gene is expressed. Furthermore, RNA *in situ* hybridization should be performed to determine the exact location of the expression of this gene and its role. Future work would be knocking out this gene and creating mutants to see if there are any quantifiable differences in the seed shattering.

## 5 Conclusion

We observed significant variation across major traits such as plant height, stem diameter, biomass, grain yield, and seed composition. Over three consecutive years, the variety Future 83 exhibited high biomass and grain yield, suggesting stable performance. Furthermore we observed seed shattering as a one of a major factor negatively impacting grain yield. Our study provided preliminary insight into the molecular basis of flowering and seed development. By comparing homologs of the soybean *Pod dehiscence 1 (GmPdh1)* and *Terminal flower 1b* ***(*** *GmTFL1b or Dt1* ***)*** genes in industrial hemp, we explored a novel aspect of hemp genetics. Further research should explore the molecular functions of these genes for validation. Additionally, future efforts should focus on producing hemp varieties with uniform growth and maturation, higher grain yield, and improved seed composition. This could be achieved by investigating the gene-environment interactions (G×E) to assess their adaptive suitability across the diverse agroecological zones of the Midwest. Overall, our findings will contribute to hemp breeding and crop improvement and support hemp farmers by enhancing cultivation practices.

## Data Availability

The original contributions presented in the study are publicly available. This data can be found here: https://doi.org/10.6084/m9.figshare.30307579.v1.

## References

[B1] Aboul-Maaty N. A. F. Oraby H. A. S. (2019). Extraction of high-quality genomic DNA from different plant orders applying a modified CTAB-based method. Bull. Natl. Res. Cent 43, 25. doi: 10.1186/s42269-019-0066-1

[B2] Alonso-Esteban Torija-Isasa M.E. de Cortes Sánchez-Mata M. (2022). Mineral elements and related antinutrients, in whole and hulled hemp (*Cannabis sativa* L.) seeds. J. Food Composition Anal. 109, 104516. doi: 10.1016/j.jfca.2022.104516 34891087

[B3] Aluko R. E. (2017). “Hemp seed (Cannabis sativa L.) proteins: Composition, structure, enzymatic modification, and functional or bioactive properties,” in Sustainable protein sources. Eds. Nadathur S. R. Wanasundara J. P. D. Scanlin &L. (Academic Press, New York, USA ), 121–132. doi: 10.1016/B978-0-12-802778-3.00007-X

[B4] Amaya I. Ratcliffe O. J. Bradley D. J. (1999). Expression of CENTRORADIALIS (CEN) and CEN-like genes in tobacco reveals a conserved mechanism controlling phase change in diverse species. Plant Cell 11, 1405–1417. doi: 10.1105/tpc.11.8.1405, PMID: 10449576 PMC144298

[B5] Asiamah J. Y. Haruna M. S. Tamang K. R. Carson C. B. Koirala P. Reed E. A.. (2025). Genome editing in grain legumes for food security. Front. Genome Editing 7. doi: 10.3389/fgeed.2025.1572292, PMID: 40474866 PMC12140438

[B6] Callaway J. C. (2004). Hempseed as a nutritional resource: An overview. Euphytica 140, 65–72. doi: 10.1007/s10681-004-4811-6

[B7] Campbell B. J. Berrada A. F. Hudalla C. Amaducci S. McKay J. K. (2019). Genotype × Environment interactions of industrial hemp cultivars highlight diverse responses to environmental factors. Agrosystems Geosciences Environ. 2, 1–11. doi: 10.2134/age2018.11.0057

[B8] Campiglia E. Radicetti E. Mancinelli R. (2017). Plant density and nitrogen fertilization affect the agronomic performance of industrial hemp (*Cannabis sativa* L.) in Mediterranean environment. Ind. Crops Products 100, 246–254. doi: 10.1016/J.INDCROP.2017.02.022

[B9] Cattaneo C. Givonetti A. Leoni V. Guerrieri N. Manfredi M. Giorgi A.. (2021). Biochemical aspects of seeds from *Cannabis sativa* L. plants grown in a mountain environment. Sci. Rep. 11, 3927. doi: 10.1038/s41598-021-83290-1, PMID: 33594196 PMC7887209

[B10] Cheng X. Wang R. Liu X. Zhou L. Dong M. Rehman M.. (2022). Effects of light spectra on morphology, gaseous exchange, and antioxidant capacity of industrial hemp. Front. Plant Sci. 13. doi: 10.3389/fpls.2022.937436, PMID: 35720586 PMC9201404

[B11] Cornell Hemp (2020). 2020 Cornell Hemp trials for New York State: grain, dual purpose, and fiber production (Geneva, New York: Cornell University Extension). Available online at: https://bpb-us-e1.wpmucdn.com/blogs.cornell.edu/dist/a/7491/files/2021/05/2020HempGrain-FiberTrialReport_2021.03.17.pdf.

[B12] Darby H. (2021). 2021 industrial grain hemp variety trial (Burlington, Vermont: UVM Extension).

[B13] Das L. Li W. Dodge L. A. Stevens J. C. Williams D. W. Hu H.. (2020). Comparative evaluation of industrial hemp cultivars: agronomical practices, feedstock characterization, and potential for biofuels and bioproducts. ACS Sustain. Chem. Eng. 8, 6200–6210. doi: 10.1021/acssuschemeng.9b06145

[B14] Deguchi M. Kane S. Potlakayala S. George H. Proano R. Sheri V.. (2020). Metabolic engineering strategies of industrial hemp (*Cannabis sativa* L.): A brief review of the advances and challenges. Front. Plant Sci. 11. doi: 10.3389/fpls.2020.580621, PMID: 33363552 PMC7752810

[B15] Deguchi M. Potlakayala S. Spuhler Z. George H. Sheri V. Agili R.. (2021). Selection and validation of reference genes for normalization of qRT-PCR data to study the cannabinoid pathway genes in industrial hemp. PloS One 16, e0260660. doi: 10.1371/journal.pone.0260660, PMID: 34928958 PMC8687539

[B16] Dhoubhadel S. P. (2021). Challenges, opportunities, and the way forward for the U.S. hemp industry Vol. 19 (Western Economics Forum, Western Agricultural Economics Association, California). Available online at: https://ideas.repec.org/a/ags/weecfo/315938.html (Accessed 2021).

[B17] Dowling C. A. Shi J. Toth J. A. Quade M. A. Smart L. B. McCabe P. F.. (2024). A FLOWERING LOCUS T ortholog is associated with photoperiod-insensitive flowering in hemp (Cannabis sativa L.). Plant J. 119, 383–403. doi: 10.1111/tpj.16769, PMID: 38625758

[B18] Downs R. J. Borthwick H. A. (1956). Effects of photoperiod on the growth of trees. Botanical Gazette 117, 310–326. doi: 10.1086/335918

[B19] ElSohly M. A. Radwan M. M. Gul W. Chandra S. Galal A. (2017). “Phytochemistry of *Cannabis sativa* L,” in Phytocannabinoids. Progress in the Chemistry of Organic Natural Products, vol. 103 . Eds. Kinghorn A. Falk H. Gibbons S. Kobayashi J. (Springer, Cham). doi: 10.1007/978-3-319-45541-9_1, PMID: 28120229

[B20] Ferfuia C. Zuliani F. Danuso F. Piani B. Cattivello C. Dorigo G.. (2021). Performance and stability of different monoecious hemp cultivars in a multi-environments trial in North-Eastern Italy. Agronomy 11, 1424. doi: 10.3390/agronomy11071424

[B21] Funatsuki H. Suzuki M. Hirose A. Inaba H. Yamada T. Hajika M.. (2014). Molecular basis of a shattering resistance boosting the global dissemination of soybeans. Proc. Natl. Acad. Sci. 111, 17797–17802. doi: 10.1073/pnas.1417282111, PMID: 25468966 PMC4273335

[B22] Gülizar KurtoğluA. Erdal İbrahim Tosun İsmail (2025). Phytoremediation potential of industrial hemp grown in sewage sludge: Growth performance and environmental impact. J. Environ. Chem. Eng. 13, 2213–3437. doi: 10.1016/j.jece.2025.117173

[B23] Hall J. Bhattarai S. P. Midmore D. J. (2012). Review of flowering control in industrial hemp. J. Natural Fibers 9, 23–36. doi: 10.1080/15440478.2012.651848

[B24] Hanano S. Goto K. (2011). Arabidopsis TERMINAL FLOWER1 is involved in the regulation of flowering time and inflorescence development through transcriptional repression. Plant Cell 23, 3172–3184. doi: 10.1105/tpc.111.088641, PMID: 21890645 PMC3203435

[B25] Hofhuis H. Moulton D. Lessinnes T. Routier-Kierzkowska A. L. Bomphrey R. J. J. Mosca G.. (2016). Morphomechanical innovation drives explosive seed dispersal. Cell 166, 222–233. doi: 10.1016/J.CELL.2016.05.002, PMID: 27264605 PMC4930488

[B26] Horska I. (2005). Edestin-comprising agent for substitution of blood plasma and a method of its production (WO2005058332A3) (World Intellectual Property Organization, Czech Republic). Available online at: https://patents.google.com/patent/WO2005058332A3/en (Accessed June 30, 2005).

[B27] House J. D. Neufeld J. Leson G. (2010). Evaluating the quality of protein from hemp seed (*Cannabis sativa* L.) products through the use of the protein digestibility-corrected amino acid score method. J. Agric. Food Chem. 58, 11801–11807. doi: 10.1021/JF102636B, PMID: 20977230

[B28] Huang J. Li Y. Shi Y. Wang L. Zhou Q. Huang X. (2019). Effects of nutrient level and planting density on population relationship in soybean and wheat intercropping populations. PloS One 14, e0225810. doi: 10.1371/journal.pone.0225810, PMID: 31790485 PMC6886861

[B29] Islam S. Hasan B. (2025). An overview of the effects of water and moisture absorption on the performance of hemp fiber and its composites. SPE Polym 6. doi: 10.1002/pls2.10167

[B30] Kempton R. A. Gleeson A. C. (1997). “Unreplicated trials,” in Statistical methods for plant variety evaluation. Plant breeding series 3 statistical methods for plant variety evaluation. Eds. Kempton R. A. Fox P. N. Cerezo M. (Springer, Dordrecht). doi: 10.1007/978-94-009-1503-9_6

[B31] Leckie K. M. Sawler J. Kapos P. MacKenzie J. O. Giles I. Baynes K.. (2024). Loss of daylength sensitivity by splice site mutation in Cannabis pseudo-response regulator. Plant J. 118, 2020–2036. doi: 10.1111/tpj.16726, PMID: 38525679

[B32] Liu M. Toth J. A. Childs M. Smart L. B. Abbaspourrad A. (2023). composition and functional properties of hemp seed protein isolates from various hemp cultivars. J. Food Sci. 88. doi: 10.1101/2022.06.01.494437, PMID: 36694405

[B33] Liu B. Watanabe S. Uchiyama T. Kong F. Kanazawa A. Xia Z.. (2010). The soybean stem growth Habit Gene Dt1 Is an ortholog of Arabidopsis TERMINAL FLOWER1. Plant Physiol. 153, 198–210. doi: 10.1104/pp.109.150607, PMID: 20219831 PMC2862436

[B34] Lynch R. C. Padgitt-Cobb L. K. Garfinkel A. R. Knaus A. R. Hartwick B. J. Allsing N. T. (2025). Domesticated cannabinoid synthases amid a wild mosaic cannabis pangenome. Nature 643, 1001–1010. doi: 10.1038/s41586-025-09065-0, PMID: 40437092 PMC12286863

[B35] Ma H. (2005). Molecular genetic analyses of microsporogenesis and microgametogenesis in flowering plants. Annu. Rev. Plant Biol. 56, 393–434. doi: 10.1146/annurev.arplant.55.031903.141717, PMID: 15862102

[B36] Maity A. Lamichaney A. Joshi D. C. Bajwa A. Subramanian N. Walsh M.. (2021). Seed shattering: A trait of evolutionary importance in plants. Front. Plant Sci. 12. doi: 10.3389/fpls.2021.657773, PMID: 34220883 PMC8248667

[B37] Marsh J. I. Nestor B. J. Petereit J. Tay Fernandez C. G. Bayer P. E. Batley J.. (2023). Legume-wide comparative analysis of pod shatter locus PDH1 reveals phaseoloid specificity, high cowpea expression, and stress responsive genomic context. Plant J. 115, 68–80. doi: 10.1111/tpj.16209, PMID: 36970933

[B38] Mishchenko S. Mokher J. Laiko I. Burbulis N. Kyrychenko H. Dudukova S. (2017). Phenological growth stages of hemp (*Cannabis sativa* L.): codification and description according to the BBCH scale. Agricultural Sciences, Lithuanian Academy of Sciences, 24. doi: 10.6001/zemesukiomokslai.v24i2.3496

[B39] Mohamedikbal S. Al-Mamun H. A. Marsh J. I. Upadhyaya S. Danilevicz M. F. Nguyen H. T.. (2024). Local haplotyping reveals insights into the genetic control of flowering time variation in wild and domesticated soybean. Plant Genome 17, e20528. doi: 10.1002/tpg2.20528, PMID: 39510980 PMC11628924

[B40] Muedi H. T. H. Kujoana T. C. Shai K. Mabelebele M. Sebola N. A. (2024). The use of industrial hemp (*Cannabis sativa*) on farm animal’s productivity, health, and reproductive performance: a review. Anim. Production Sci. 64, AN23268. doi: 10.1071/AN23268

[B41] National Agricultural Statistics Service (2022). National hemp report 2022. Available online at: https://www.nass.usda.gov/Publications/Todays_Reports/reports/hempan22.pdf.

[B42] National Oceanic and Atmospheric Administration (2023). (NOAA: National Oceanic and Atmospheric Administration. U.S. Department of Commerce). Available online at: https://www.noaa.gov/ (Accessed July 13, 2025).

[B43] Osborne T. B. (1924). The Vegetable Proteins. 2nd ed (London: Longmans, Green and Co).

[B44] Petit J. Salentijn E. M. J. Paulo M. J. Denneboom C. Trindade L. M. (2020a). Genetic architecture of flowering time and sex determination in hemp (*Cannabis sativa* L.): A genome-wide association study. Front. Plant Sci. 11. doi: 10.3389/fpls.2020.569958, PMID: 33250906 PMC7672029

[B45] Petit J. Salentijn E. M. J. Paulo M.-J. Thouminot C. van Dinter B. J. Magagnini G.. (2020b). Genetic variability of morphological, flowering, and biomass quality traits in hemp (*Cannabis sativa* L.). Front. Plant Sci. 11. doi: 10.3389/fpls.2020.00102, PMID: 32153610 PMC7044243

[B46] Podder S. Shafian S. Thomason W. E. Wilson T. B. Fike J. H. (2024). Hemp seed yield responses to nitrogen fertility rates. Crops 4, 145–155. doi: 10.3390/crops4020011

[B47] Poudel S. Poudel B. Acharya B. Kathayat D. Pant K. Tamang K. R.. (2020). Effect of crop establishment methods on performance of rice in Rupandehi, Nepal. J. Institute Agric. Anim. Sci. 36. doi: 10.3126/jiaas.v36i1.48391

[B48] Quach T. N. Nguyen H. T. M. (2015). Valliyodan, B. et al. Genome-wide expression analysis of soybean NF-Y genes reveals potential function in development and drought response. Mol. Genet. Genomics 290, 1095–1115. doi: 10.1007/s00438-014-0978-2, PMID: 25542200 PMC4435856

[B49] Rahemi A. Dhakal R. Temu V. W. Rutto L. Kering M. K. (2021). Performance of different-use type industrial hemp cultivars under mid-atlantic region conditions. Agronomy 11, 2321. doi: 10.3390/agronomy11112321

[B50] Stack G. M. Toth J. A. Carlson C. H. Cala A. R. Marrero-González M. I. Wilk R. L.. (2021). Season-long characterization of high-cannabinoid hemp (*Cannabis sativa* L.) reveals variation in cannabinoid accumulation, flowering time, and disease resistance. GCB Bioenergy 13, 546–561. doi: 10.1111/gcbb.12793

[B51] Sun X. Sun Y. Li Y. Wu Q. Wang L. (2021). Identification and characterization of the seed storage proteins and related genes of *cannabis sativa* L. Front. Nutr. 8. doi: 10.3389/fnut.2021.678421, PMID: 34164425 PMC8215128

[B52] Tang K. Wang J. Yang Y. Deng G. Yu J. Hu W.. (2022). Fiber hemp (Cannabis sativa L.) yield and its response to fertilization and planting density in China. Ind. Crops Products 177, 114542. doi: 10.1016/J.INDCROP.2022.114542

[B53] Tong H. Jin Y. Liu W. Li F. Fang J. Yin Y.. (2009). DWARF AND LOW-TILLERING, a new member of the GRAS family, plays positive roles in brassinosteroid signaling in rice. Plant J. 58, 803–816. doi: 10.1111/j.1365-313X.2009.03826.x, PMID: 19220793

[B54] Toth J. A. Stack G. M. Cala A. R. Carlson C. H. Wilk R. L. Crawford J. L.. (2020). Development and validation of genetic markers for sex and cannabinoid chemotype in *Cannabis sativa* L. GCB Bioenergy 12, 213–222. doi: 10.1111/gcbb.12667

[B55] Van Roekel R. J. Coulter J. A. (2011). Agronomic responses of corn to planting date and plant density. Agron. J. 103, 1414–1422. doi: 10.2134/agronj2011.0071

[B56] Vasantha Rupasinghe H. P. Davis A. Kumar S. K. Murray B. Zheljazkov V. D. (2020). Industrial Hemp (*Cannabis sativa* subsp. sativa) as an Emerging source for value-added functional food ingredients and nutraceuticals. Molecules 25. doi: 10.3390/molecules25184078, PMID: 32906622 PMC7571072

[B57] Visković J. Sikora V. Latković D. Zeremski T. Dunđerski Dušan Astatkie T.. (2024). Optimization of hemp production technology for fiber and seed. Ind. Crops Products 219, 0926–6690. doi: 10.1016/j.indcrop.2024.119127

[B58] Wang Y. Li J. (2008). Molecular basis of plant architecture. Annu. Rev. Plant Biol. 59, 253–279. doi: 10.1146/annurev.arplant.59.032607.092902, PMID: 18444901

[B59] Wickland D. P. Hanzawa Y. (2015). The FLOWERING LOCUS T/TERMINAL FLOWER 1 gene family: Functional evolution and molecular mechanisms. Mol. Plant 8, 983–997. doi: 10.1016/j.molp.2015.01.007, PMID: 25598141

[B60] Williams M. M. (2006). Planting date influences critical period of weed control in sweet corn. Weed Sci. 54, 928–933. doi: 10.1614/WS-06-005R.1

[B61] Williams A. Brym Z. Chen C. Collins A. Crawford J. Darby H.. (2025). Comparing agronomic performance of industrial hemp varieties for suitable production in the United States. Agron. J. 117. doi: 10.1002/agj2.70006

[B62] Wimalasiri E. M. Jahanshiri E. Chimonyo V. G. P. Kuruppuarachchi N. Suhairi T. A. S. T. M. Azam-Ali S. N.. (2021). A framework for the development of hemp (*Cannabis sativa* L.) as a crop for the future in tropical environments. Ind. Crops Products 172, 113999. doi: 10.1016/j.indcrop.2021.113999, PMID: 35071705 PMC8762045

[B63] Yang J. Kornet R. Diedericks C. F. Yang Q. Berton-Carabin C. C. Nikiforidis C. V.. (2022). Rethinking plant protein extraction: Albumin—From side stream to an excellent foaming ingredient. Food Structure 31, 100254. doi: 10.1016/J.FOOSTR.2022.100254

[B64] Zhang L. Bollin V. Gao I. A. P. (2025). Dissecting the molecular basis of variability for "owering time in Camelina sativa. Plant Biotechnol 23, 2290–2302. doi: 10.1111/pbi.70049, PMID: 40111913 PMC12120899

