# Supplementary material for: Phenotypic and genetic characterization of sixteen grain and dual-type industrial hemp varieties (Cannabis sativa L.) for agronomic and yield component traits
Source: Front Plant Sci. 2025 Oct 20;16:1632346. doi: 10.3389/fpls.2025.1632346 (PMC12580366; doi:10.3389/fpls.2025.1632346)
Supplement: Supplementary file 1 [file Supplementaryfile1.docx]

**Genetic Characterization of Sixteen Grain and Dual-Type Industrial Hemp Varieties (*Cannabis sativa* L.) for Agronomic and Yield Component Traits.**

Kusum Raj Tamang^1^, Thomas P. Mawhinney^2^, Christian B. Carson^1^, Joshua Asimah^1^, Sakina Mahdi^1^, Emily Reed^1^, Swastika Sharma^1^, Prabesh Koirala^1^, Jaimin Patel^3^, Clement Akotsen Mensah^4^, Babu Valliyodan**^1*^**

1. Department of Agriculture and Environmental Sciences, Lincoln University of Missouri, Jefferson City, Missouri
2. Department of Biochemistry, University of Missouri, Columbia, Missouri
3. IR-4 Project Headquarters, North Carolina State University, Raleigh, North Carolina, USA
4. Alabama Cooperative Extension System, Alabama A&M and Auburn Universities, Normal, Alabama

***Corresponding author: ValliyodanB@lincolnu.edu**

**Supplementary Materials****File 1: Overview of the experiment explaining trait selection for the study, marker development, qPCR analysis, and the experiment’s future direction particularly RNA sequencing and cloning for further.**

**
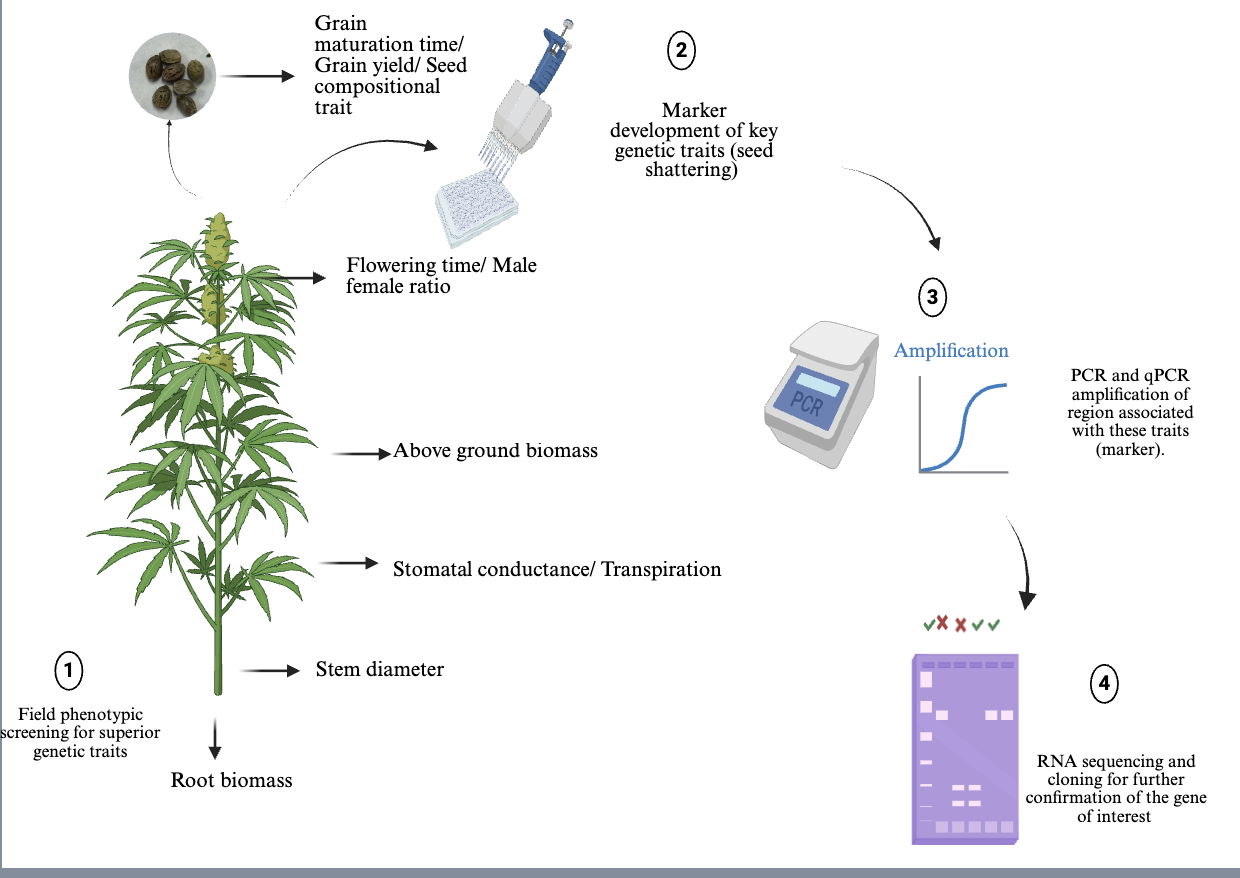
**

**File 2: This figure explains the weather conditions of the experimental site for the planting season 2022/2023/2024. The variability in air temperature, rainfall, relative humidity, soil temperature, and soil moisture is presented in the graph below.**

The distribution of average monthly mean air temperature, rainfall, relative humidity, soil moisture, and soil temperature is shown in Figure 1. The average rainfall during the planting period was highest in 2024, followed by 2023 and 2022. Similarly, relative humidity and soil moisture were also higher in 2024, followed by 2023 and 2022.


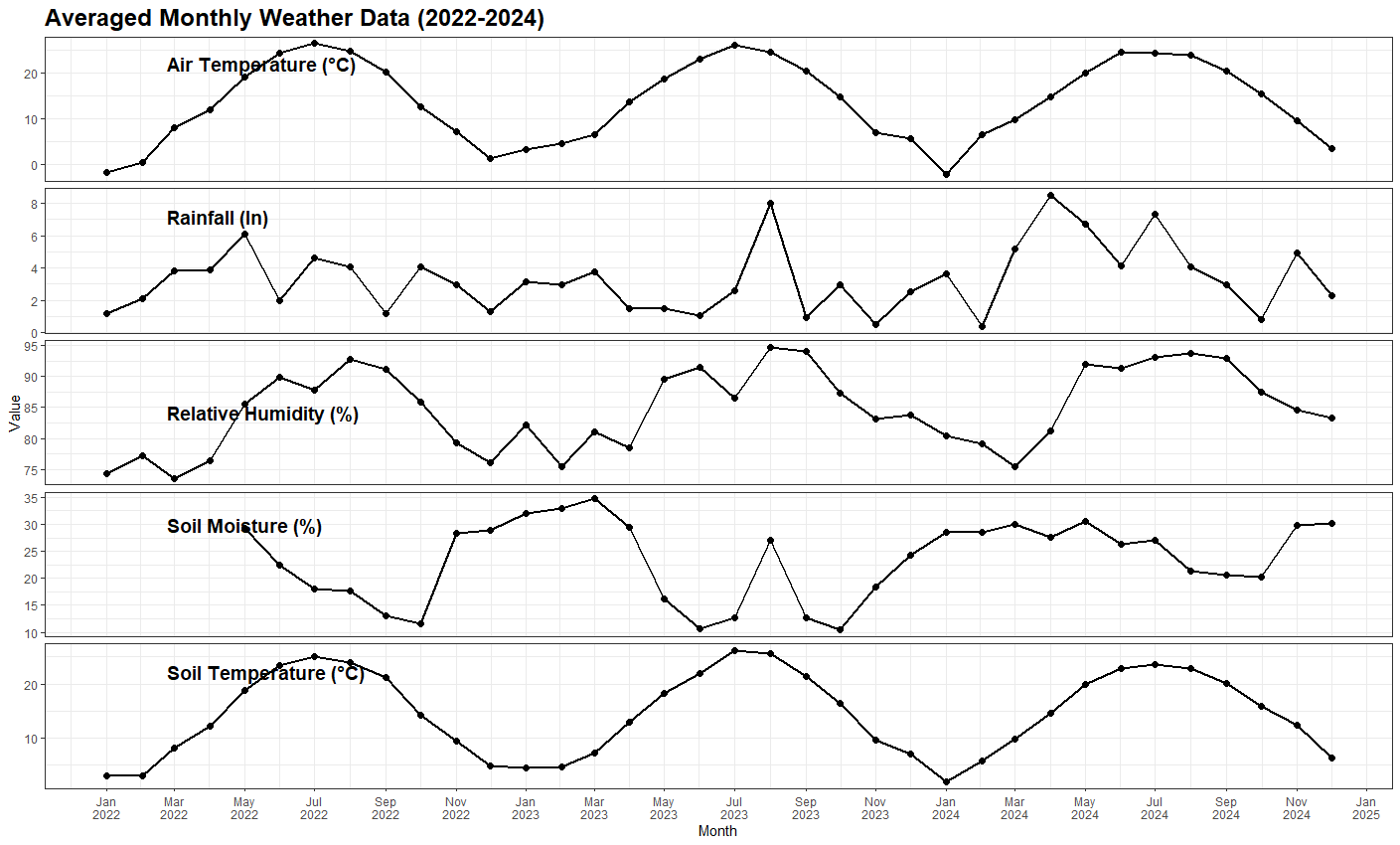


**File 3: Figure displaying the Aerial view of the experimental plot at George Washington Carver Farm, Lincoln University, Jefferson City, Missouri, 2023. Though the plot was two replications, the plot size was 10ft *100 ft each with duplication deemed to capture maximum variability.**

**
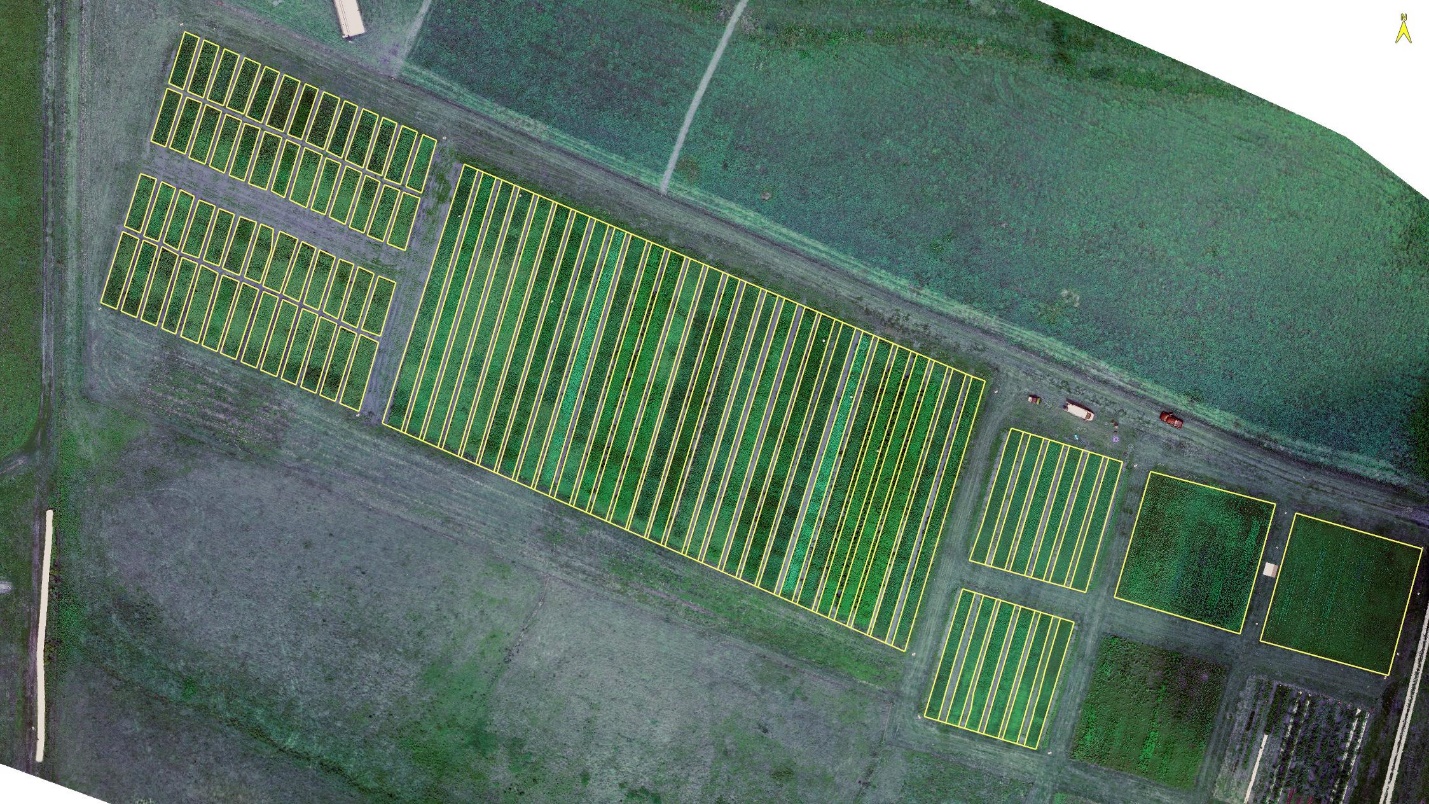
**

**.**

**File 4. The figure displaying the Sexual Dimorphism in Cannabis sativa: A. Monoecious B. Dioecious Varieties, Lincoln University, Missouri. In the monoecious varieties, male and female flowers are found on the same plant, as shown on the left (A), while for the dioecious varieties, the male flowers and female flowers are found on different plants (B).**

** File 5. Table showing the days of flowering in Male plants in different hemp varieties**

B

A

| Varierty | Days to male flowering |
| --- | --- |
| Altair | NA |
| Bialobrzeskie | NA |
| BVL1 | 50 |
| BVL2 | 50 |
| BVL3 | 50 |
| Carmenecta | 50 |
| Felina | NA |
| Ferimon | NA |
| Fibror 79 | NA |
| Futura 83 | Na |
| Gravity | 55 |
| Henola | NA |
| Jinma | 90 |
| Orion | NA |
| Santhica | NA |
| Uso 31 | NA |
| Vega | NA |

**File 6: Developing positive and negative controls for the CsDrrp2 gene. Positive and negative controls were developed for relative gene expression, and the graph presented below provides evidence of the suitability of the housekeeping gene and the negative control tissues. The figure shows the Expression of the positive and negative controls. The positive control used was the TUB α3, while the negative control was the root tissue. Figure B shows the gradient qPCR run of CsCENLP explaining the annealing temperature used in the experiment.**

**File 7: Figures displaying the different stages in seed development and shattering. Figure A displays the physiological mechanism in *Cannabis sativa*. Figure B shows various stages in seed development. Each seed is surrounded by a single bract with an abscission zone that opens with seed maturation. The following figure shows the tissues at the collection stages, and the respective RNA was extracted for the expression analysis.**


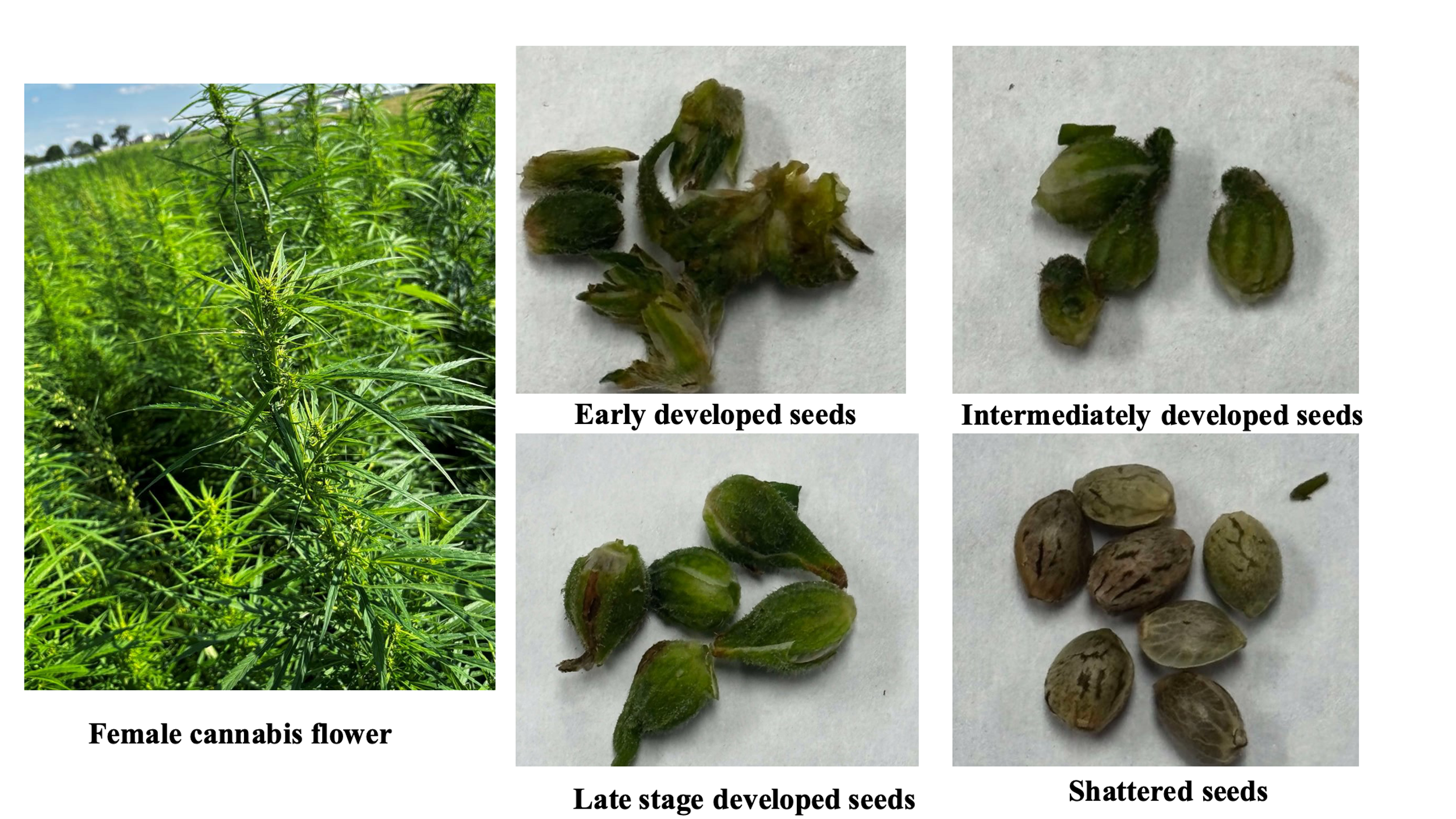


B

A

**File 8: Figure illustrating the regression analysis between the whole plant biomass and the stem diameter, final plant height, shoot biomass, and root biomass, which are the factors governing the whole plant biomass. We ran multiple linear regression analyses for whole plant biomass against other key traits that can influence this character. We found that stem diameter, final plant height, shoot biomass, and root biomass had a strong relationship with the whole plant biomass, with the R-squared value of greater than 70% for all the parameters**.

**File 9: *The graph showing the root/shoot ration of the plant at the time of harvest; the varieties represented by the different letters are significantly different from each other; the bar graph indicated by the same color represent the same group based on the Tukey HSD test at 5 % level of significance; the scoring (0-10) was done based on visual observation of the plants***


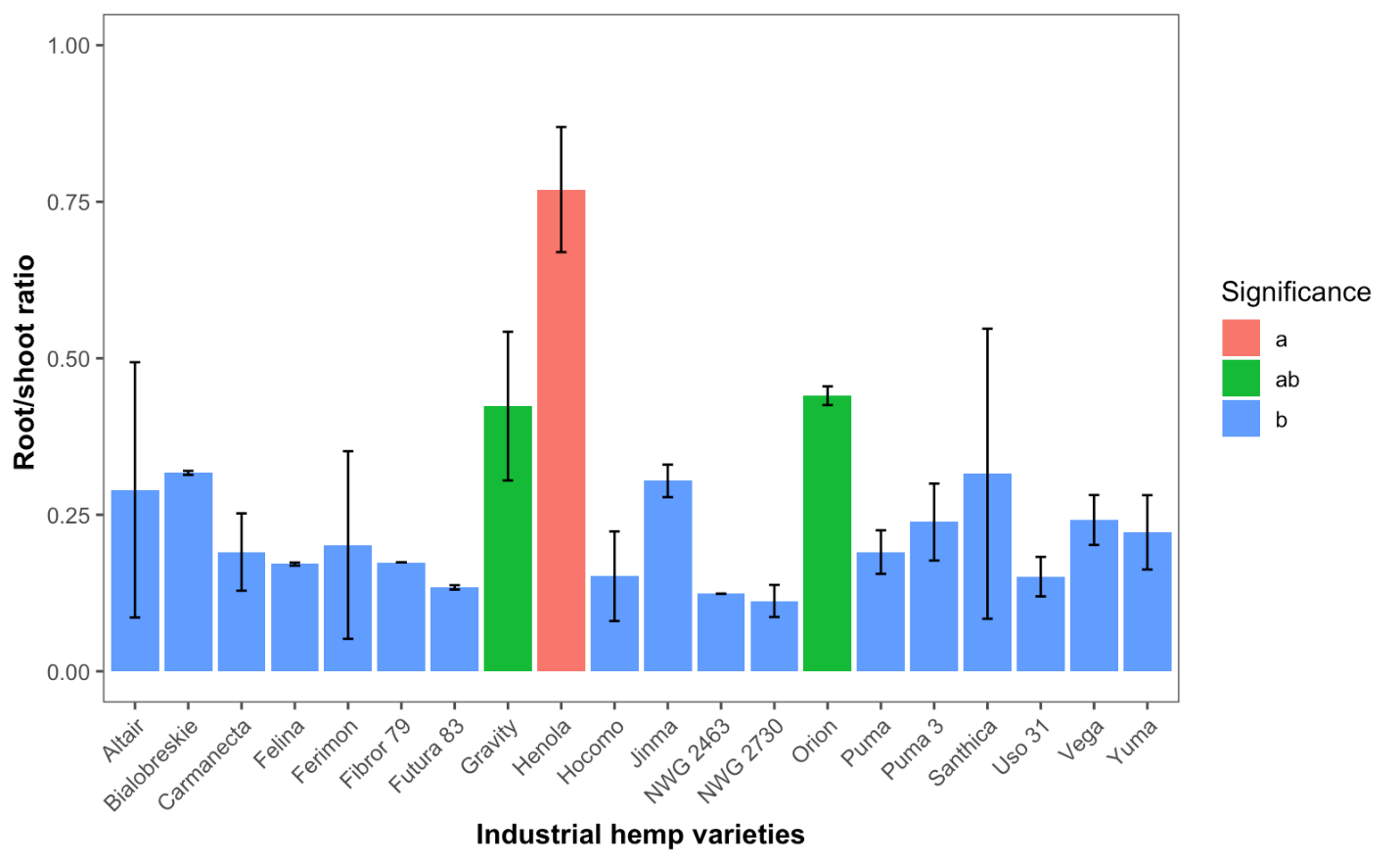


File 10. This table presents the results of the analysis of variance (ANOVA), evaluating the influence of different years and the interaction between year and variety on the yield trait. It provides statistical evidence of temporal and genotype-by-environment interaction effects on yield performance.

| **Source** | **Df** | **Sum Sq** | **Mean Sq** | **F value** | **Pr(>F)** |  |
| --- | --- | --- | --- | --- | --- | --- |
| TRT | 2 | 9762562 | 4881281 | 67.908 | 9.57e-06 *** | |
| Year | 2 | 508944 | 254472 | 3.54 | 0.0792 . |  |
| REP | 1 | 712 | 712 | 0.01 | 0.9232 |  |
| TRT:Year | 4 | 225745 | 56436 | 0.785 | 0.5658 |  |
| Residuals | 8 | 575047 | 71881 |  |  |  |
|  |  |  |  |  |  |  |

**File 11. The graph showing the parameters that contribute to maximum variability in the PCA biplot.**


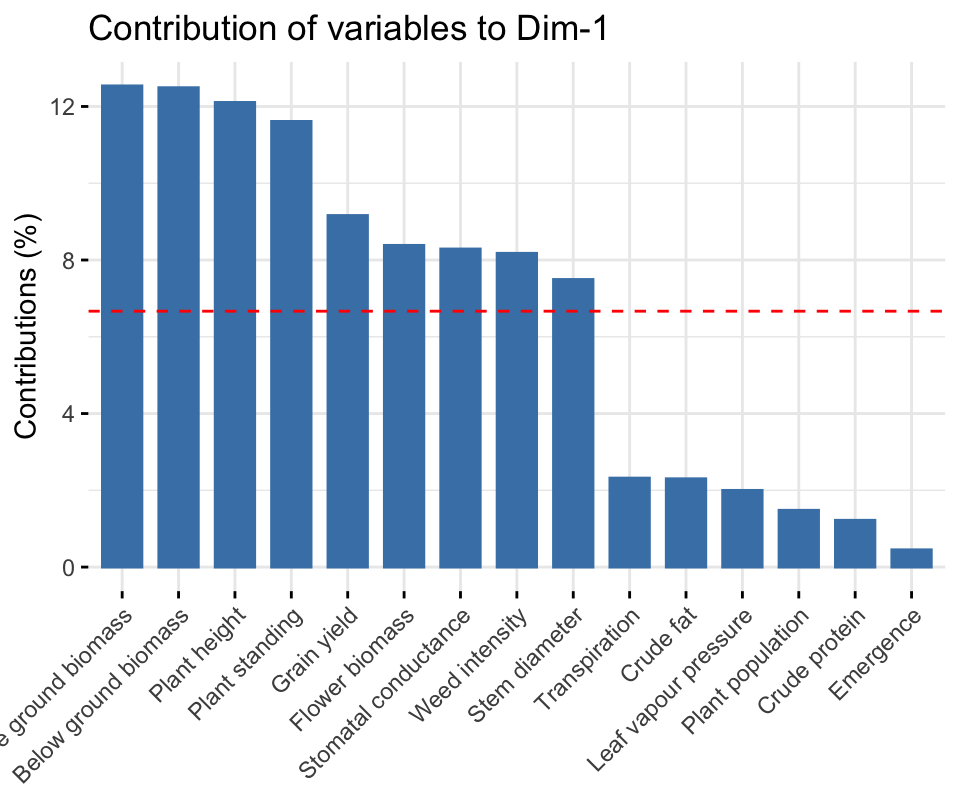

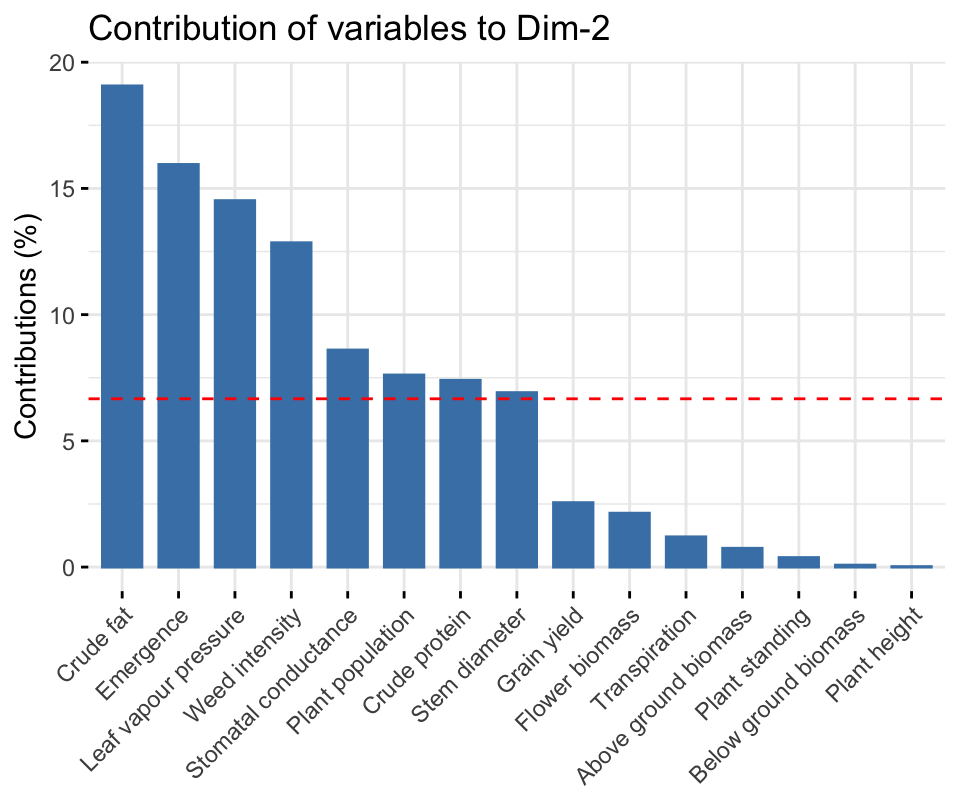

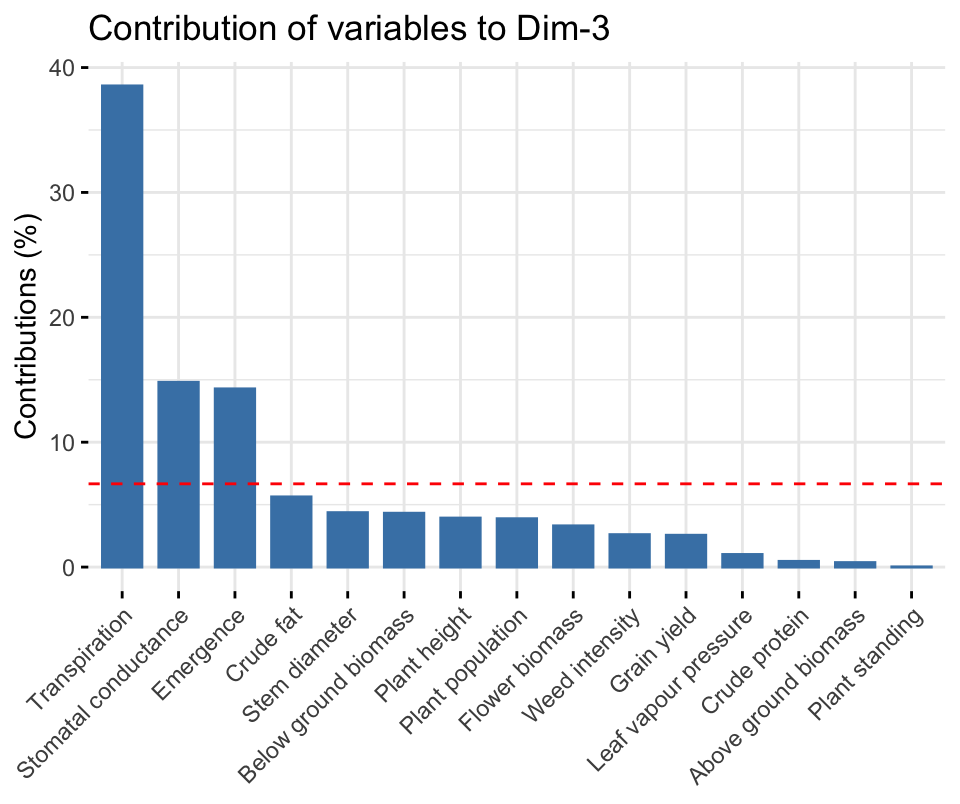


File 12: A gradient PCR was performed to determine the optimal annealing temperature for CsCENLP2 amplification. Although the PCR product was clearly visible at 50 °C, this temperature was used as a reference point, and further optimization was conducted. A standardized annealing temperature was later used during qPCR to ensure accurate and reproducible results.
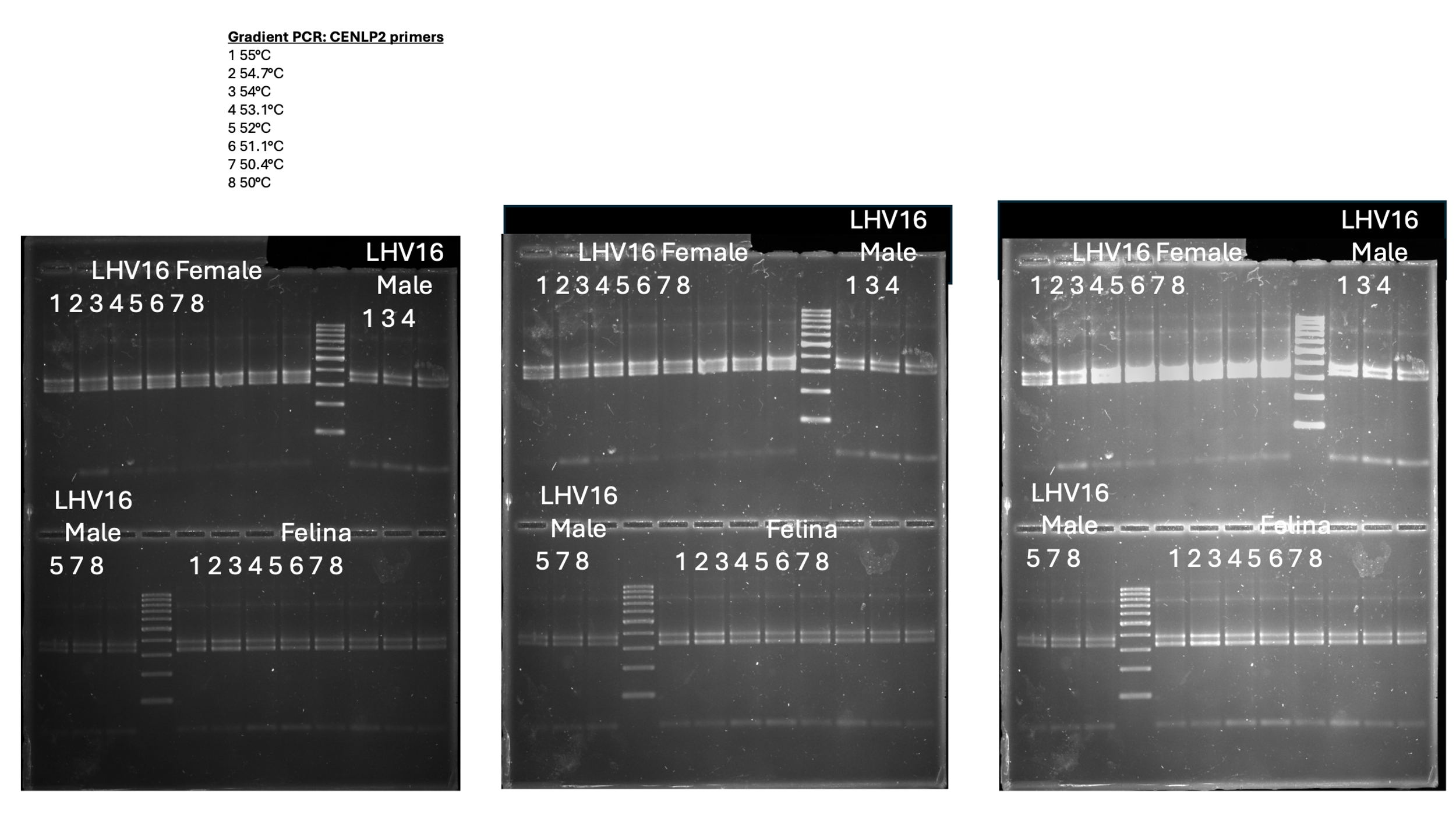


**File 13: Figure describing the** Tissue specific expression of CsDrrp2 in the early flowering stage in Futura 83.


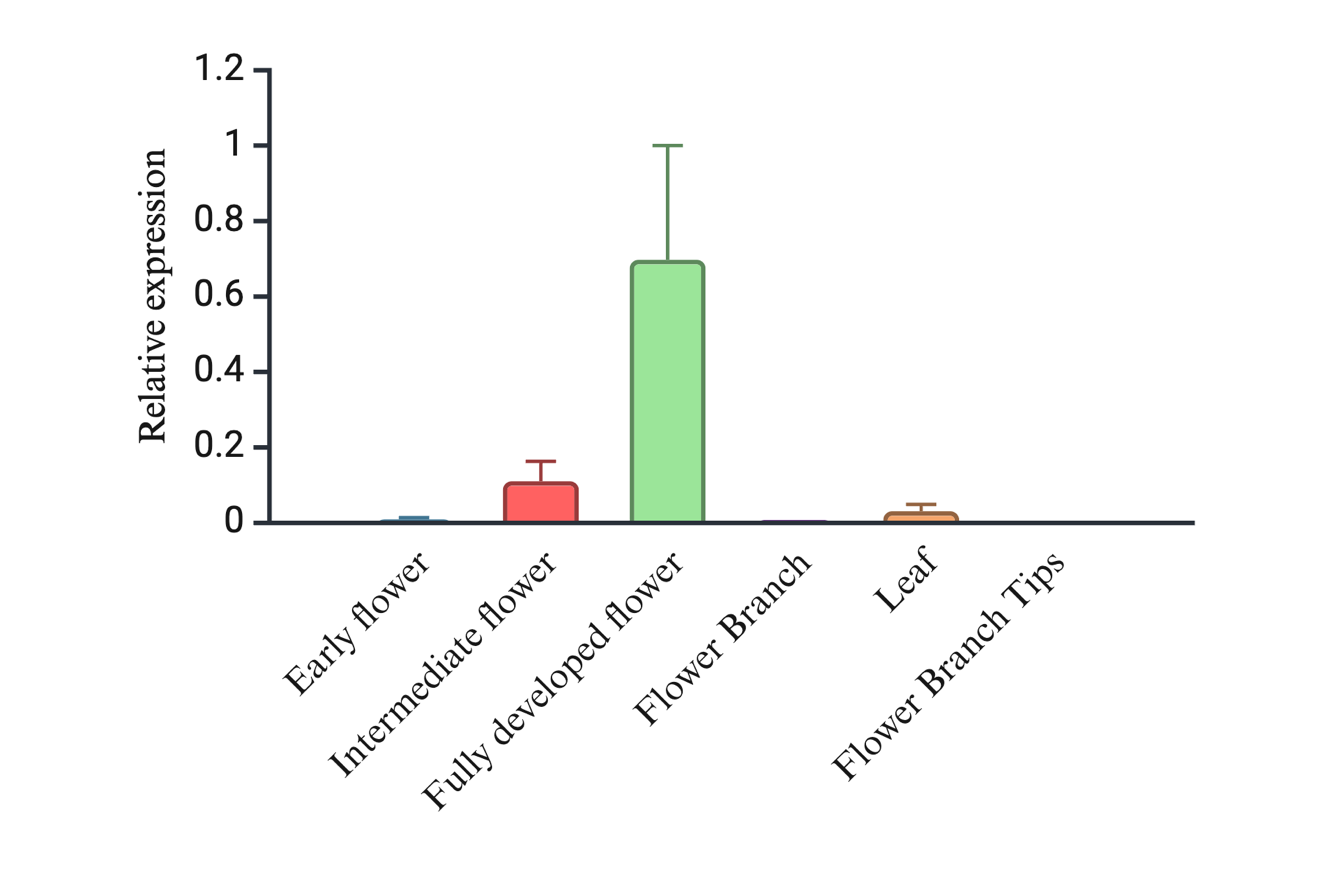


**File 14: ANOVA summary table for *CsDrrp2* gene expression across tissues.**
This summary table presents the ANOVA results for *CsDrrp2* expression across different tissue types. Although variation in expression levels was observed among tissues, the differences were not statistically significant, indicating no strong tissue-specific expression pattern under the tested conditions.

| **Df** | **Sum Sq** | **Mean Sq** | **F value** | **Pr(>F)** |
| --- | --- | --- | --- | --- |
| 4 | 0.708201 | 0.17705 | 5.392963 | 0.065749 |
| 1 | 0.057912 | 0.057912 | 1.764007 | 0.25484 |
| 4 | 0.131319 | 0.03283 |  |  |

Signif. codes: 0 ‘***’ 0.001 ‘**’ 0.01 ‘*’ 0.05 ‘.’ 0.1 ‘ ’ 1

**File 15: ANOVA summary corresponding to Figure 6A of the manuscript.**
This table summarizes the ANOVA results for *CsDrrp2* gene expression in different tissues of the LHV-16 variety. Although expression levels varied across tissues, the differences were not statistically significant.

| **Df** | **Sum Sq** | **Mean Sq** | **F value** | **Pr(>F)** |
| --- | --- | --- | --- | --- |
| 1 | 1326527 | 1326527 | 0.519414 | 0.482173 |
| 15 | 54419771 | 3627985 | 1.420571 | 0.252412 |
| 15 | 38308373 | 2553892 |  |  |
|  |  |  |  |  |

Signif. codes: 0 ‘***’ 0.001 ‘**’ 0.01 ‘*’ 0.05 ‘.’ 0.1 ‘ ’ 1

File 16: A**NOVA summary corresponding to Figure 6B of the manuscript.**
This table presents the ANOVA results for *CsDrrp2* gene expression across different tissues of the Futura 83 variety. While variation in expression was observed among tissues, the differences were not statistically significant.

| **Df** | **Sum Sq** | **Mean Sq** | **F value** | **Pr(>F)** |
| --- | --- | --- | --- | --- |
| 6 | 1.565593 | 0.260932 | 19.2226 | 1.66E-05 |
| 2 | 0.032027 | 0.016013 | 1.179693 | 0.340622 |
| 12 | 0.162891 | 0.013574 |  |  |
|  |  |  |  |  |

Signif. codes: 0 ‘***’ 0.001 ‘**’ 0.01 ‘*’ 0.05 ‘.’ 0.1 ‘ ’ 1

**File 17** **ANOVA summary for *CsCENLP2* gene expression in the Futura 83 variety.**
This table shows the ANOVA results for *CsCENLP2* expression across different tissues of the Futura 83 variety. Although expression levels varied, the differences were not statistically significant under the tested conditions.

| **Df** | **Sum Sq** | **Mean Sq** | **F value** | **Pr(>F)** |
| --- | --- | --- | --- | --- |
| 4 | 0.178436 | 0.044609 | 181.8965 | 6.93E-08 |
| 2 | 0.005331 | 0.002666 | 10.86978 | 0.005236 |
| 8 | 0.001962 | 0.000245 |  |  |
|  |  |  |  |  |

**File 18: Convex hull categorization of the PCA data** ****

File 19: Delta 9 THC among the tested varieties


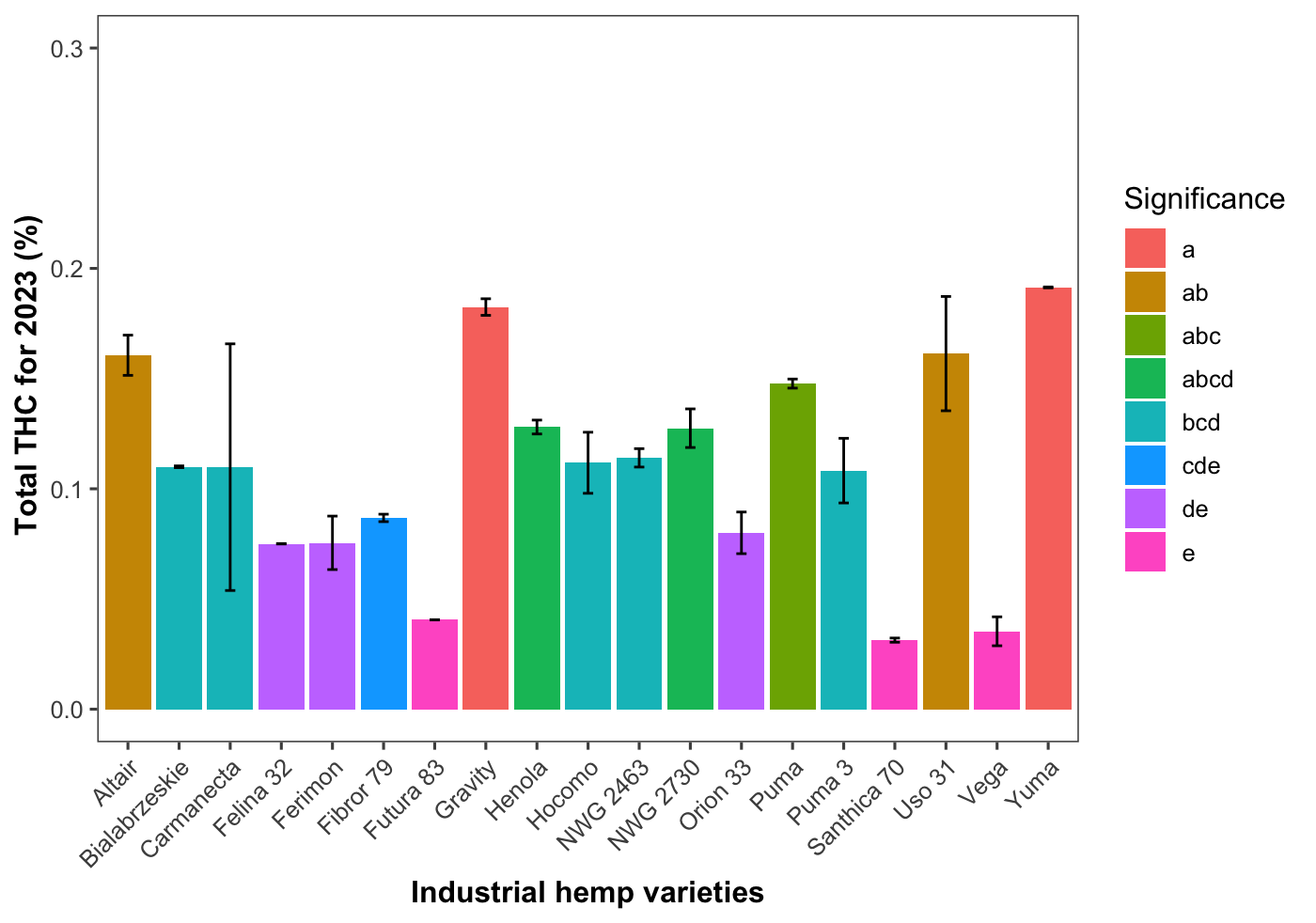


File 20. Average shattering scores based on visual observation

Golden Kush (Cannabinoid type)-1.5

Jinma-2.5

LHV-17-4.5

Note: While this shattering score provides visual estimate, this study should be complemented with the additional verification such as lignin deposition, torsion force and temperature effect.

**File 21: Sources of the hemp seeds:**

| **S.N** | **Variety** | **Source** |
| --- | --- | --- |
| 1 | Altair | Hemppoint |
| 2 | Bialobrzeskie | Internationhemp.com |
| 3 | Felina | Hempoint |
| 4 | Ferimon | Hempoint |
| 5 | Orion | Hempoint |
| 6 | Uso 31 | Internation-hemp.com |
| 7 | Vega | Horizon hemp |
| 8 | BVL1 | Lincoln University_ landrace collection |
| 9 | BVL2 | Lincoln University_ landrace collection |
| 10 | Jinma | Individual contact |
| 11 | Henola | Individual contact |
| 12 | Carmenecta | Internatinal-hemp.com |
| 13 | Fibror 79 | Individual contact |
| 14 | Futura 83 | KonopiUS |
| 15 | Gravity | Individual contact |
| 16 | BVL3 | Lincoln University_ landrace collection |
| 17 | Santhica | Hemp point |
