# Supplementary material for: Phenotypic and genetic characterization of sixteen grain and dual-type industrial hemp varieties (Cannabis sativa L.) for agronomic and yield component traits
Source: Front Plant Sci. 2025 Oct 20;16:1632346. doi: 10.3389/fpls.2025.1632346 (PMC12580366; doi:10.3389/fpls.2025.1632346)
Supplement: Supplementary file 2 [file Supplementaryfile2.docx]

**Gene expression protocol of the homolog of the soybean pod dehiscence gene (Gm Pdh1) and Cs CEN-like protein 2 in dual-type industrial hemp and wild-type industrial hemp.**

**Step 1. RNA extraction**

RNA was extracted using modified Trizole method. More extraction protocol could be found in the original MS.

**Step 2: RNA purification and DNAse treatment**

RNA purification and DNase treatment were critical steps in preparing high-quality RNA for qPCR-based gene expression studies. These steps ensured the accuracy and reliability of the qPCR results. After RNA was extracted, it was quantified using a NanoDrop spectrophotometer. Each sample was then treated with DNase to remove residual genomic DNA that could compromise the accuracy of the experiment. The DNAse treatment protocol could be provided upon request. The RNA was calculated again after DNASe treatment.

**Step 3: cDNA synthesis**

We calculated the total RT enzyme required by multiplying the number of samples by 1.5 µL and verified that the available RT enzyme was sufficient for the experiment. The total 5X Buffer needed was calculated using the formula:
5X Buffer required = Number of samples × 6 µL,
and we ensured there was enough buffer available. The RNA sample concentration was 200 ng/ul.

For each 200 ng/ul RNA sample, we prepared the reaction mix as follows:

- Added 0.38 µL of 2 µM reverse primer,
- Added 1.5 µL of dNTPs,
- Added the required volume of nuclease-free water to bring the final reaction volume to 22.5 µL.

To streamline the process, we prepared a cocktail containing the reverse primer, dNTPs, and water in appropriate proportions.

The incubation steps were carried out as follows:

- Tubes were incubated at 25°C for 10 minutes,
- Followed by incubation at 50°C for 15 minutes,
- The reaction was terminated by heating at 85°C.

We also ensured that all tubes were sealed properly to prevent evaporation or contamination during the reaction.

.

**Step 4: qPCR reaction (provided in MS)**

**Prepare the cocktail of primer, PCR master mix, and water:**

For each reaction, add:

- - **1.5 µL** of the cDNA template
  - **1.5 µL** of the forward and reverse primers (2 µM each)
  - **7.5 µL** of the PCR master mix
  - **4.5 µL** of water

**Reaction conditions:**

- The annealing temperature for the **CsDrrp2** gene was maintained at **47°C**.The gradient annealing temperature gel figure provided.
- The annealing temperature for the **CsCENLP** gene was maintained at **47°C**.
- The method selected for the qPCR reaction was **4-step amplification endpoint, 15 µL total reaction volume**.

**Step 5: Running the gel:**

5 ul of the sample was taken and 10 ul of the 0.5 TBE was taken for the total volume of 15 ul. 1/6^th^ volume of 6X dye the solution was added. 2.13% Agarose gel was prepared for running the gel.
