# Supplementary material for: Phenotypic and genetic characterization of sixteen grain and dual-type industrial hemp varieties (Cannabis sativa L.) for agronomic and yield component traits
Source: Front Plant Sci. 2025 Oct 20;16:1632346. doi: 10.3389/fpls.2025.1632346 (PMC12580366; doi:10.3389/fpls.2025.1632346)
Supplement: Supplementary file 3 [file Supplementaryfile3.docx]

**>NC_044375.1:751491-753925 Cannabis sativa chromosome 2, CsCENLP cs10, whole genome shotgun sequence, Pink pepper**

12345678901234567890123456789012345678901234567890123456789012345678901234567890123456789012345678901234567890123456789

TTATCGTTTTCTGCATGCAGTTTCTCTTTGAGCGTTGAAGAAAAGAGCAGCAACAGGAGGACCAAGGTCATTTTGGGTAGCAAAGAGGCGCGTGTTGAAACGCTCTCTCGATGAAGGTGGCATTACCACTGTTTGTCTTCTCTTCTGTTTAAACATTAAGAACACAAACCTATGTATTCCTATGTTTGGTCGTGGCATTTCATAGCTCACTATCTCTTCTCCTATAATATATTAATTCATTTAAACATTAATTATTCTCATAATTGATTAAAAAATTAAAAACATATACATATTTACTATATAATAATTACCAAATGACACATCAGTTGTTCCTGGAATGTCTGTCACTATCCTGCAATTATTTCAACAACAAATATTAATTTCAACTCAAATTAATTTAGAAACTCATACCATTTTAAATTTTGCCTTTTGCTTAAGTTATTGATTAAAATCTCTATTTTGTTAAATTATAAATTGGATTATGCATTTTTCAAAATAGTATAAAATATAGTATATATATATTCTGATTGGCTGATTTTTTGACAAGAAAAACTCAATAATAATTTGATCTATAGGTATTACGACAAGACTGATTATATTTTCTGTATCTATTTGTGTTAAAAATTATCTTCAAGTTGGTTATATTAAAAATTATCAAAAATTGAGTTCAAAATGCTAAAGTGTACTATTTTAGAAAATGCAGGATCCAATTCATTATTTAACAAAACAGAAGATCCAATCGGTAAATTTTGTAAAATACAGAGTCCAACTATTATAACCCTACATAAATATATGTATATATATATATTATATATTAGAGAAAAATATTTATTACTAATTAAATTCTAGAAAGTAATATATTATTGAGAAAGAACATGTTTTCATTTCATTCAAAAGTAAAATAATATGCTAATTAGAATTTTTGCCTCCTAAAACTTTGACATGTACTAAATTATGTTTCCTGAATTTTTAAGGTCGTTAAAAATGTTCTTCGAATTATTGAGATTATTAAATTTAAAGACTTTTGTCTAATTTCATTTAATTTTACTAATTCAGTGATTGTTTATGTACTAAATTATGCTCCCCAAACTTTAATATCTACCAAATCATGCCACCTGAACTTTCCTCCATGTTAGTTTTTTTACTAAAATTAGACAAAAAATTTTAAATCCAACAATCTCAATATAATTCAGAGAACATCATTTTTAACGACCTTAAAAGTTGAAATAATATAATATAGAGTGGACACCTGATAACTAATTGTTGTATATTATTATTAATTTAATATTATTTGCTATGACTTTATAATTAATCAAAATACTTTTTTAATTATTTGTTGAAATTCTGAGATCTTGTGATGACTAATTAAGGATCATGTGTGGTCTCACCAGTTGCATATATTCCCTTAAGTATACTTATTATTATACTATTCCCCTATATATTATATTATATAAAAAAAAGAAGAATATTTATAATTATATATACATAGATTAGACATATATATTATATTAATTAATTAATAATATTAAGACCTTCGTCCCCCTAACGATCGATCAAGATGTGTACAGTGTCTCAAATTATACGACAGCTTAATTAGATGAATATAAATATTTATATTTATAATTAAAAAGTGAAAATAGCTATACACAAGTTGAATAATATTATATATAAATAAGAAATATGTATGATCGGAATTTTATAGGATAAAAAAATTAATTATCAATATTATATATAGTATTAGGTATATATTTAAATTTTTTAGTTTATATCTTTTCAATCTCTCTCTCTAGTAATAAAATAACTTATATATGAATATTATAGGTAATTAATTTTGTCTAATAATATATTTAATAATAAGTGATATAACCCAAAAACTTTGTCATCACACTTATTATATTTTAATAATCATATTAAAAAATAGTACTTAATTTTATAATATATGTAGAAGATATTTTAATTATTATTACATCAAATAAATCATGATAATTTTGCTTTAATTAATTTGTAGCAATGTATATATATAGTAATTTAATATAATTTAATTATTTTTGTAGTTACCAGTGTAAATGCTCCCTCAAATAAGGATCACTAGGGCCAGGAACATCAGGATCAGTCATCACCTAATTAATATTATTTATACAACAAAATAAACAACATTATTATTTAACAAAATGATGTATATATATATAATTAATTTAAAACATGTAATTAATTAATTAATTAATTATTACCAGTGTGAAAAAGGATCTCATGTCACCACCATGAACCTCAACTTTTGGCTTAATAGTAATTGTGGAAGGAAACAACTCATGGCCATTGTAGACTCTCTTGTTTGAGTTGTAAGTCACTGTAAATTTTAGGGTTGGAGAGAAAACATCAACCACATCTCCTATCACTCTTCCAATAATAAGAGGATTATCTGACATTCTTGCCAT

**Disease resistance response protein 206**

**>NC_044374.1:84358594-84359187 Cannabis sativa chromosome 5, cs10, whole genome shotgun sequence, Pink pepper**

TCACAACCAACACTCGTAGAGTTTAATATGAACCCTAAGTCTAAAATAAACTTCACCTTCAAAAGCATCAGTCATCAAAGTAGCAATCCCTCTTGACATGAAAAAATCTCCAGTCCCACCAACAACCGAAACATCCCTAGTTTTGTTCATAAGAGGATCAGCTCCAGCGAAGTTAAGGCTACCCTTGTGCTCAGTCGAGTTGAACACAAAGGAAAACCCTAACCAAGCTGTGAACACATCTTTCTTGTCGTACAAGTAAAACCCTTGAGCTCGGCCAACTGGGGCCGAGTGAAGATTGTTGTCGAGTGTAATGGGGTCATCGAATACTACCAAGTCCCCAAAATGGTTTTTCCCAGCTAATAGGGTTTTGTTACCCCAAGCTGGAGCTCCAACAATGGCTGAGGTTGCATTCTTGGCATTTTCTCCATTGTATATAATGTCATGGAAATAAAACACTAGACTTTTACATGGCTTAATAAGCTTATTATTTTTCATCATTGATGATGATTGGGGTAGGGCATTGTTAAAATCATTGATGAATATGATGATGAAGAAGAGGATTAGACTCAAAAATATTGGCTTGGAAGACTTCATGCAAAACCTTATATATATACCCCATGCAATTAAAAACCCTAGCTTGCATATATTTTTCCTTCCCTATATTAAATATCCAAAACATATACTAAATGAAGTCTTCCAAGCCAATATTTTTGAGTCTAATCCTCTTCTTCATCATCATATTCATCAATGATTTTAACAATGCCCTACCCCAATCATCATCAATGATGAAAAATAATAAGCTTATTAAGCCATGTAAAAGTCTAGTGTTTTATTTCCATGACATTATATACAATGGAGAAAATGCCAAGAATGCAACCTCAGCCATTGTTGGAGCTCCAGCTTGGGGTAACAAAACCCTATTAGCTGGGAAAAACCATTTTGGGGACTTGGTAGTATTCGATGACCCCATTACACTCGACAACAATCTTCACTCGGCCCCAGTTGGCCGAGCTCAAGGGTTTTACTTGTACGACAAGAAAGATGTGTTCACAGCTTGGTTAGGGTTTTCCTTTGTGTTCAACTCGACTGAGCACAAGGGTAGCCTTAACTTCGCTGGAGCTGATCCTCTTATGAACAAAACTAGGGATGTTTCGGTTGTTGGTGGGACTGGAGATTTTTTCATGTCAAGAGGGATTGCTACTTTGATGACTGATGCTTTTGAAGGTGAAGTTTATTTTAGACTTAGGGTTCATATTAAACTCTACGAGTGTTGGTTGTGATCATGATCATGATTGATCAATTTATTAATGTTTTACGATGATGATCATCACATAAAAATAAATTAATGTGATCATCAAGTTATTAATAATTTGTTACATTTAATTATGATGATTTGTTATTATTTTTACTTTTTTTTGTCATTTTTCTTTTTGGGTTTGGTGTTAATTGGTCGTTTGTTTTTTACTTTTTGATGATTAAGATTTCTAATCATCGTGTGCTGAAAATAATTTCTAGTGTTATTAGAAAGTGTGATCATTATTTATTAAATATGTTATATATATATATGAATCGATTATCCATTACCATAAAATAAATAATTAATTAAATGGTCAAATATAATAATATATAATCTGAAGTTATGAGCTTGCAAAACCTTATATATATACCCCATGCAATTAAAAACCCTAGCTTGCATATATTTTTCCTTCCCTATATTAAATATCCAAAACATATACTAAATGAAGTCTTCCAAGCCAATATTTTTGAGTCTAATCCTCTTCTTCATCATCATATTCATCAATGATTTTAACAATGCCCTACCCCAATCATCATCAATGATGAAAAATAATAAGCTTATTAAGCCATGTAAAAGTCTAGTGTTTTATTTCCATGACATTATATACAATGGAGAAAATGCCAAGAATGCAACCTCAGCCATTGTTGGAGCTCCAGCTTGGGGTAACAAAACCCTATTAGCTGGGAAAAACCATTTTGGGGACTTGGTAGTATTCGATGACCCCATTACACTCGACAACAATCTTCACTCGGCCCCAGTTGGCCGAGCTCAAGGGTTTTACTTGTACGACAAGAAAGATGTGTTCACAGCTTGGTTAGGGTTTTCCTTTGTGTTCAACTCGACTGAGCACAAGGGTAGCCTTAACTTCGCTGGAGCTGATCCTCTTATGAACAAAACTAGGGATGTTTCGGTTGTTGGTGGGACTGGAGATTTTTTCATGTCAAGAGGGATTGCTACTTTGATGACTGATGCTTTTGAAGGTGAAGTTTATTTTAGACTTAGGGTTCATATTAAACTCTACGAGTGTTGGTTGTGATCATGATCATGATTGATCAATTTATTAATGTTTTACGATGATGATCATCACATAAAAATAAATTAATGTGATCATCAAGTTATTAATAATTTGTTACATTTAATTATGATGATTTGTTATTATTTTTACTTTTTTTTGTCATTTTTCTTTTTGGGTTTGGTGTTAATTGGTCGTTTGTTTTTTACTTTTTGATGATTAAGATTTCTAATCATCGTGTGCTGAAAATAATTTCTAGTGTTATTAGAAAGTGTGATCATTATTTATTAAGATTTATTTGTTCAGATGCTTTCAAAAAAAAAAATTTAATAACTTTGATTATTCCAACGAAAGAACAAAAGCTTTTAATATTAATTAATGTTTGCACCAATATTTGTACAAGTATTTTTGGTAATTTGTGGCATAAATATTTAAATTTAACTTCTAATGATAATTATATTTAAATTATATATTTAGAACTTTAGTTCATA
